# Robust RNAi enhancement via human Argonaute-2 overexpression from plasmids, viral vectors and cell lines

**DOI:** 10.1093/nar/gkt836

**Published:** 2013-09-17

**Authors:** Kathleen Börner, Dominik Niopek, Gabriella Cotugno, Michaela Kaldenbach, Teresa Pankert, Joschka Willemsen, Xian Zhang, Nina Schürmann, Stefan Mockenhaupt, Andrius Serva, Marie-Sophie Hiet, Ellen Wiedtke, Mirco Castoldi, Vytaute Starkuviene, Holger Erfle, Daniel F. Gilbert, Ralf Bartenschlager, Michael Boutros, Marco Binder, Konrad Streetz, Hans-Georg Kräusslich, Dirk Grimm

**Affiliations:** ^1^Department of Infectious Diseases, Virology, Heidelberg University Hospital, Im Neuenheimer Feld 324, D-69120 Heidelberg, Germany, ^2^Cluster of Excellence CellNetworks, Heidelberg University, Im Neuenheimer Feld 267, D-69120 Heidelberg, Germany, ^3^Department of Medicine III, University Hospital Aachen, Pauwelstrasse 30, D-52074 Aachen, Germany, ^4^Department of Infectious Diseases, Molecular Virology, Heidelberg University, Im Neuenheimer Feld 345, D-69120 Heidelberg, Germany, ^5^Division Signaling and Functional Genomics, German Cancer Research Center, Im Neuenheimer Feld 580, D-69120 Heidelberg, Germany, ^6^BioQuant, Heidelberg University, Im Neuenheimer Feld 267, D-69120 Heidelberg, Germany and ^7^Department of Pediatric Oncology, Hematology, Immunology and Pulmonology, Heidelberg University Hospital, Im Neuenheimer Feld 350, D-69120 Heidelberg, Germany

## Abstract

As the only mammalian Argonaute protein capable of directly cleaving mRNAs in a small RNA-guided manner, Argonaute-2 (Ago2) is a keyplayer in RNA interference (RNAi) silencing via small interfering (si) or short hairpin (sh) RNAs. It is also a rate-limiting factor whose saturation by si/shRNAs limits RNAi efficiency and causes numerous adverse side effects. Here, we report a set of versatile tools and widely applicable strategies for transient or stable Ago2 co-expression, which overcome these concerns. Specifically, we engineered plasmids and viral vectors to co-encode a codon-optimized human Ago2 cDNA along with custom shRNAs. Furthermore, we stably integrated this Ago2 cDNA into a panel of standard human cell lines via plasmid transfection or lentiviral transduction. Using various endo- or exogenous targets, we demonstrate the potential of all three strategies to boost mRNA silencing efficiencies in cell culture by up to 10-fold, and to facilitate combinatorial knockdowns. Importantly, these robust improvements were reflected by augmented RNAi phenotypes and accompanied by reduced off-targeting effects. We moreover show that Ago2/shRNA-co-encoding vectors can enhance and prolong transgene silencing in livers of adult mice, while concurrently alleviating hepatotoxicity. Our customizable reagents and avenues should broadly improve future *in vitro* and *in vivo* RNAi experiments in mammalian systems.

## INTRODUCTION

One and a half decades after its discovery in nematodes (1), RNA interference (RNAi) has become a widely used surrogate genetic tool with great clinical potential (2). Part of its attraction stems from the fact that typical RNAi triggers—small interfering (si) or short hairpin (sh)RNAs—use cellular molecules (miRNAs) as blueprints and thereby tap into evolutionarily conserved endogenous pathways. Compared with artificial gene silencers, such as antisense oligonucleotides or synthetic ribozymes, this mimicry of natural processes is not only more effective but was initially also believed to invoke fewer side effects. Increasing evidence suggests, however, that this presumed benefit can turn into a severe disadvantage. This was first illustrated in 2006 by findings that shRNA-induced RNAi in livers of adult mice can cause dose-dependent morbidity and mortality (3). The ensuing model that excessive shRNA expression had nonspecifically overwhelmed the cells’ RNAi capacity was recently supported by similar *in vivo* shRNA cytotoxicities in various organs in mice, rats and dogs, occasionally also resulting in fatalities (4–9). In parallel, numerous *in vitro* studies consistently demonstrated that multiple si or shRNAs can compete with each other when delivered or expressed simultaneously, further backing up the concept that eukaryotic RNAi mechanisms are saturable (10–12).

A common notion in many of these reports as well as in an associated meta-analysis (13) was that cytotoxicity or competition correlated with a perturbation of endogenous miRNA biogenesis or functionality. This implied that one or a few components of the RNAi pathway, which are engaged by si, sh and miRNAs, are rate-limiting and thus titratable (4,13–15). To narrow down these factors, we and others inhibited or overexpressed different proteins involved in RNAi and characterized the effects on co-delivered si or shRNAs *in vitro* and *in vivo* (3,10,11,16–20). One of the hits was Exportin-5, the karyopherin shuttling mi and shRNA precursors from the nucleus to the cytoplasm (3,18–21). However, even more restrictive and thus critical is Argonaute-2 (Ago2) (11,16,17), a core component of the RNA-induced silencing complex (RISC). In mammals, Ago2 is one of four orthologs (Ago1–4) that share the ability to load small RNA duplexes and to activate them by removing one of the two strands (the passenger strand), resulting in RISC carrying a single-stranded (ss) guide RNA. The latter directs RISC to an mRNA, which is then cleaved, destabilized and/or translationally inhibited, largely depending on the degree of its complementarity to the guide RNA. Notably, Ago2 is unique among the four orthologs in that it is the only mammalian Ago protein that can directly cleave or slice mRNAs (hence Ago2’s alternative name Slicer) owing to the RNase H-like activity of its PIWI domain (22–25). This intrinsic slicing ability also plays a crucial role during the biogenesis of certain cellular miRNAs, as evidenced by reduced baseline expression of mature miRNAs in Ago2 knockout cells (26,27), or Ago2-cleaved precursor (ac-pre-)miRNAs and Ago2-dependent Dicer-independent processing of miR-451 (26,28–30). Moreover, only Ago2 can slice and thus effectively remove the passenger strand of a loaded perfect RNA duplex, explaining its superior ability to activate thermodynamically stable si or shRNAs, in contrast to the other three Ago orthologs, which rely on poorly characterized strand displacement mechanisms (31,32). Recently, the Berkhout lab has exploited this distinctive Ago2 property by designing a novel minimal shRNA variant (‘AgoshRNAs’) that circumvents Dicer and is instead loaded directly into Ago2, which will process and then use it as a guide to cleave target mRNAs (33). Last but not least, the essential and unique role of Ago2 is further highlighted by findings that Ago2, but not Ago1, 3 or 4, knockouts are lethal in mice (23).

As a whole, these exclusive features readily explain why Ago2 is a central factor in si- or shRNA-mediated gene silencing, and why disrupting its natural functions by abundant accumulation of ectopic RNAi triggers is detrimental for the cell. This concurrently suggests that attempts to increase Ago2 concentration and thus overcome its rate-limiting nature should result in broadly applicable new avenues to improve *in vitro* and *in vivo* gene silencing applications. We and others have previously provided evidence that Ago2 co-delivery *in trans* can indeed enhance si or shRNA efficiencies (16,17). Yet, the need for a separate Ago2 expression construct has hampered the routine use of this approach, and especially its translation into high-throughput concepts or into *in vivo* RNAi applications. Here, we implemented and comprehensively evaluated three improved strategies to boost the potency of si and shRNAs through Ago2 overexpression from (i) plasmids or (ii) viral vectors that co-encode an shRNA, or from (iii) human cell lines, which can be transfected or infected with any RNAi molecule. We show that all three avenues can significantly increase target mRNA knockdowns by different classes of RNAi triggers, and that these Ago2-induced enhancements concurrently translate into more robust and more reproducible RNAi phenotypes. Importantly, we also provide evidence that long-term Ago2 expression does not adversely perturb endogenous gene or miRNA expression, but rather can reduce RNAi off-targeting activities. Lastly, we demonstrate that Ago2/shRNA co-expression from bi-cistronic viral vectors can alleviate *in vivo* toxicities and provide more sustained inhibition of a hepatic transgene in adult mice. As all three strategies are highly versatile, readily customizable and compatible with existing RNAi triggers, our new tools and principles should find wide applications in future *in vitro* or *in vivo* RNAi experiments.

## MATERIALS AND METHODS

### Cell culture

Huh7 and Huh7.5 (human hepatoma cells), 293T (human embryonic kidney cells) and HeLaP4 [a derivative of HeLa (human cervical carcinoma cells) expressing the human immunodeficiency virus (HIV)-1 CD4 and CXCR4 receptors (34)] cells were grown in Dulbecco's modified Eagle's medium (DMEM; Invitrogen/Gibco, Darmstadt, Germany) supplemented with 10% fetal calf serum (FCS) (Biochrom AG, Berlin, Germany), 200 µM l-glutamine (Invitrogen/Gibco) and penicillin-streptomycin (Invitrogen/Gibco). Transfections with plasmid DNA or siRNAs were carried out in 96-well (for luciferase assays), 24-well (for western blot analyses) or 10-cm dishes (for generation of stable cell lines), using Lipofectamine 2000 (Invitrogen GmbH, Karlsruhe, Germany) and following the manufacturer's instructions for each format.

### Plasmids and siRNAs

*Renilla* and Firefly luciferase were co-expressed from plasmid psiCheck2 (Promega, Mannheim, Germany). Plasmids encoding a 1.3-fold hepatitis B virus (HBV) genome (pTHBV2), or the murine p53 or human alpha-1-antitrypsin (hAAT) cDNAs were reported before (3,35).

For transient or stable Ago2 expression, we created a set of constructs encoding a codon-optimized Ago2 cDNA variant that we had previously adapted for maximum expression in human cells (17). Details of all cloning steps described in the following are depicted in Supplementary Figure S1. The parental plasmid for all constructs has been published and encodes Flag/HA-tagged human wild-type Ago2 cDNA under a cytomegalovirus (CMV) promoter together with a neomycin resistance cassette (Addgene plasmid 10822) (24). In a first step, we replaced the wild-type Ago2 cDNA with the abovementioned codon-optimized variant as a NotI/EcoRI fragment. The resulting plasmid pCA2n was used in all transient transfection experiments as well as for generation of stable Ago2-expressing Huh7.5 cells (see below). For stable integration into 293T cells, which already contain a neomycin resistance gene, we replaced the latter in pCA2n with a blasticidin resistance gene [amplified from plasmid pWPI-blr (36) using as primers Blfor 5′ AATCCC **CCCGGG** ATGGCCAAGCCTTTGTCTCAAG 3′ and Blrev 5′ CCCTAG **TCTAGA** TTAGCCCTCCCACACATAAC 3′; XmaI (Blfor) and XbaI (Blrev) sites used for cloning are in bold]. The resulting plasmid pCA2b was also used for selection in HeLaP4 cells.

To facilitate creation of plasmids based on pCA2n expressing both shRNAs and Ago2, we aimed for a basic construct permitting simple shRNA insertion as annealed oligonucleotides behind a U6 promoter. However, we could not directly adapt our prior strategy (3) because the enzyme (BbsI) used to linearize our original U6 promoter-containing plasmid already cut the Ago2-encoding recipient pCA2n construct three times. We therefore re-amplified the U6 promoter via polymerase chain reaction (PCR) using as primers U6for 5′ ACCG **CTCGAG** CGAGTCCAACACCCGTGG 3′ and U6rev 5′ ACCG **CTCGAG** AAAAAAT ***GAGACG*** AGTGAAGC ***CGTCTC*** CGGTGTTTCGTCCTTTCCACAAG 3′, and cloned the product as XhoI fragment (XhoI sites in bold) into the unique XhoI site in pCA2n. The reverse primer introduced two BsmBI restriction sites (bold/italics), which replaced the former BbsI sites and likewise allowed directional cloning of annealed shRNA oligonucleotides into the new plasmid pCA2nU6 (example in Supplementary Figure S1B).

In parallel, the same XhoI U6 fragment was cloned into a control plasmid encoding Yfp instead of Ago2 (Addgene plasmid 10 825; the distributor's Web site (www.addgene.org) incorrectly reports this plasmid to express Gfp), resulting in construct pCYnU6. As for plasmid pCA2nU6, shRNAs were then cloned as annealed oligonucleotides into BsmBI-linearized pCYnU6.

Plasmids pCA2b, pCA2nU6 and pCYnU6 were next used as templates to create Adeno-associated viruses (AAV) vector constructs likewise co-expressing shRNA and Ago2 or Yfp, respectively. As recipient vector, we used a derivative of the AAV plasmid pSSV9 in which two inverted terminal repeats (ITRs) from AAV serotype 2 (AAV2) flank the AAV2 *rep* and *cap* genes (37), and into which we had previously introduced an additional SpeI site close to the 3′ITR. In a first step, we PCR-amplified a CMV-Ago2 fragment [lacking the polyadenylation (polyA) site] from pCA2b using the primers CMV_BamHI_fw 5′ TTTT **GGATCC** ACATTGATTATTGACTAGTT 3′ and Ago2_EcoRI_rev 5′ TTTT **GAATTC** TCATCAGGCGAAGTACATGG 3′, yielding a CMV-Ago2 fragment flanked with BamHI and EcoRI sites (bold). Moreover, we PCR-amplified a second fragment comprising the U6 promoter, the two BsmBI cloning sites, the T6 terminator sequence (a stretch of six thymidines serving as terminator signal for the U6 promoter) as well as the bovine growth hormone polyA signal, from pCYnU6 using the primers BGH_EcoRI_fw 5′ TTTT **GAATTC** CGCTGATCAGCCTCGACTGTG 3′ and U6_AscI_XbaI_rev 5′ TTTT **TCTAGA** GGCGCGCC GCGAGTCCAACACCCGTGGG 3′. This yielded a PCR product in which the entire U6-BsmBI-T6-polyA fragment was flanked by EcoRI and XbaI sites (shown in bold in the primers). We next performed a triple ligation in which the two PCR products were first cut with the indicated enzymes and then simultaneously ligated into BglII/SpeI-linearized pSSV9, producing the final plasmid pAAVCA2U6.

In addition, we generated a control plasmid pAAVCYU6 containing Yfp instead of Ago2, using a similar three fragment strategy. Briefly, we first PCR-amplified a CMV-Yfp fragment (also lacking a polyA signal) from pCYnU6 using the primers CMV_SalI_fw 5′ TTTT **GTCGAC** ACATTGATTATTGACTAGTTATTAATAG 3′ and YFP_EcoRI_rev 5′ TTTT **GAATTC** TTAAAGCTTCTTGTACAGCTCG 3′. To ensure that the entire ITR-flanked insert in the final pAAVCYU6 construct would later be comparable in size to the insert in pAAVCA2U6, we furthermore amplified a 1.8-kb noncoding stuffer fragment from the psiCheck2 vector using the primers stuffer_SpeI_fw 5′ TTTT **ACTAGT**AGGATGGCACCGCTGGCGAGC 3′ and stuffer_XhoI_rev 5′ TTTT **CTCGAG** CACCTCCCCCTGAACCTG 3′. Enzymes used to subsequently cut the two PCR products are indicated in the primer names, and their recognition sites are highlighted in bold. We then again performed a triple ligation to insert the two digested PCR fragments into SpeI/EcoRI-cut pAAVCA2U6, resulting in replacement of the CMV-Ago2 cassette with the stuffer-CMV-Yfp fragment.

As for the plasmids above, we also used the unique BsmBI sites for straight-forward and directed insertion of shRNA-encoding oligonucleotides into the pAAVCA2U6 or pAAVCYU6 vector plasmids.

The following shRNAs were reported before: luc29, sAg19, sAg25, hAAT19, hAAT25 (numbers denote stem lengths in nucleotides) as well as the five anti-p53 shRNAs used in Figure 4F (3,17,35). In addition, we cloned six different shRNAs against *Renilla* luciferase using our previously reported scheme (3) with the following sense strands (all in 5′-3′ direction): Ren1 GCA ACG CAA ACG CAT GAT CAC, Ren2 GCT GGA CTC CTT CAT CAA C, Ren3 GGC CTT TCA CTA CTC CTA CGA, Ren4 GCC TGA CAT CGA GGA GGA TAT, Ren5 GGA GGA TAT CGC CCT GAT CAA and Ren6 GCT ATT GTC GAG GGA GCT AAG. Detection of shRNA expression and processing via northern blotting was essentially performed as described recently (25).

The lentiviral plasmid pWPI-Ago2-BLR was generated by cloning the codon-optimized Ago2 into pWPI-BLR (36). Briefly, the Ago2 cDNA was isolated from pIRES-Ago2-U6 via NotI/EcoRI digestion, blunted using the large Klenow fragment and then ligated into PmeI/SmaI-linearized pWPI-BLR.

For infection-based luciferase reporter assays (see below), we generated and used a ss AAV vector co-expressing *Renilla* and Firefly luciferase. Therefore, a 4.45-kb fragment containing both expression cassettes [SV40 promoter-driven *Renilla* luciferase (hRluc) and a thymidine kinase promoter-driven Firefly luciferase (hluc+)] was transferred from psiCheck2 into the pSSV9 AAV vector context. This fragment was amplified using as primers pSSV9_psi_BglII_fw 5′ TTTT **AGATCT** GCGCAGCACCATGG 3′ and pSSV9_psi_SpeI_rev 5′ TTTT **ACTAGT** TTATCGATTTTACCACATTTGTAGAG 3′. The resulting PCR product was flanked by BglII and SpeI sites (bold) and was subsequently ligated into BglII/SpeI-linearized pSSV9, yielding the final plasmid pAAVpsi2.

### siRNAs

The siRNAs used in the VSV-G assays targeting human α-COP (SI00351491), CLTC (SI00299880) or GM130 (SI04235147), as well as the nonsilencing control siRNA ‘All Stars’ (SI03650318) were purchased from Qiagen (Hilden, Germany). The siRNAs used in the hepatitis C virus (HCV) assays were from the following commercial sources: ONT#1 (negative control; Dharmacon, D-001810-01), CD81 [inhibits entry of HCV, custom design from MWG/Operon (Ebersberg, Germany)], antisense strand (UUGUCUCCCAGCUCCAGAUtt), PIP4K (inhibits HCV replication; Ambion, s224264), VPS35 (38) (Qiagen, SI04268131), HCV321 (targets HCV genome within the 5′ nontranslated region (NTR), custom design from MWG/Operon, antisense strand UGCACGGUCUACGAGACCUtt). The siRNAs used in the HIV-1 infection experiment in Figure 5 against PLK1 (s448), FASN (s5030), MYO10 (s9223) or PAK4 (s20134), as well as the negative control NS#1, were purchased from Applied Biosystems (Foster City, CA, USA).

### Generation of stable cell lines

To generate stable Ago2 cell lines via plasmid transfection, cells were grown in 10-cm dishes to ∼90% confluence and transfected with 24 µg of Ago2 expression plasmids pCA2n (Huh7.5) or pCA2b (293T and HeLaP4) using Lipofectamine 2000. For selection and maintenance of stably transfected cells, blasticidin (293T and HeLaP4) (MoBiTec, Göttingen, Germany) or G418 (Huh7.5) (Invitrogen/Gibco) were added to the culture media to final concentrations of 5 µg/ml (blasticidin) or 500 µg/ml (G418), respectively. To generate stable Ago2 cell lines via lentiviral transduction, 293T, A549 or SupT1 cells were infected with viral vectors derived from plasmid pWPI-AGO2-BLR and subsequently selected with blasticidin. Briefly, lentiviral particles were produced as described (39) by calcium phosphate transfection of three individual plasmids at a 3:1:3 ratio into 293T cells: (i) pCMV-ΔR8.91, coding for HIV Gag-Pol; (ii) pMD.2G, encoding the VSV-G glycoprotein; and (iii) the lentiviral vector pWPI-AGO2-BLR or empty pWPI-BLR. pCMV-ΔR8.91 and pMD.2G were kind gifts from Didier Trono, Lausanne(40). Cell-free supernatants were harvested 48, 56 and 72 h after transfection and used for three subsequent target cell transductions (8–12 h each). Successfully transduced cells were selected by supplementing the culture medium with 5 µg/ml blasticidin after the last transduction round.

### AAV vector production

AAV vectors were produced using a standard protocol involving transfection of 293T cells with equal amounts of an AAV helper plasmid (encoding AAV *rep* and *cap* genes), an AAV vector plasmid [encoding the transgene(s)] and an adenoviral helper construct. The basic plasmids and the general protocol were reported (41). The AAV helper plasmids used here either encoded the AAV2 (for infection of standard cell lines) or AAV8 (for *in vivo* infections) capsid gene or a novel synthetic capsid sequence called AAV1P4 that permits superior infection of many cell types and that will be described in detail elsewhere. The AAV vector plasmids were either based on the conventional pSSV9 construct whose packaging results in ss DNA-containing AAV particles (37); this comprises all constructs co-expressing shRNA and Ago2 or Yfp, respectively. Alternatively, they were derived from an optimized self-complementary AAV vector genome containing two inverted copies of the same transgene that we reported before (3).

For vector production, ten 15-cm^2^ dishes with 4 × 10^6^ 293T cells per dish were seeded two days before transfection. The cells were then triple-transfected with 14.6 µg each of adenoviral helper, AAV helper and AAV vector plasmid (numbers are for one dish) using polyethylenimine (PEI) as transfection reagent. Briefly, the DNA (43.8 μg in total) was diluted in DMEM without any supplements, mixed well with 140 µl of PEI (1 mg/ml in H_2_O), and incubated for at least 30 min at room temperature. The mixture was subsequently added dropwise to the cells and incubated at 37°C for 3 days. The cells were then scraped into the medium and centrifuged at 1200 rpm for 15 min, washed with 1× phosphate buffered saline (PBS) and transferred to 50-ml falcon tubes. Next, the pellet was resuspended in 6 ml of lysis buffer (50 mM Tris–HCl, pH 8.5, 50 mM NaHCO_3_) and subjected to five freeze-thaw cycles (−80/37°C). The cell lysate was incubated with 50 U benzonase per ml for 1 h at 37°C, before cell debris was spun down at 3750 rpm for 20 min. For virus purification, the lysate was added to a preformed gradient of 15, 25, 40 and 60% iodixanol (OptiPrep in PBS-MK; 1× PBS with 1 mM MgCl_2_, 2.5 mM KCl) and centrifuged in a 70.1 Ti rotor (Beckman Coulter) at 50 000 rpm and 4°C for 2 h. Purified viruses were finally retrieved from the 40% iodixanol phase using needle and syringe, and stored in 50 µl of aliquots at −80°C. Virus titers were determined as vector genome copy numbers per ml via standard real-time PCR (RT-PCR).

### AAV infections of cells and mice

At day 0, 3000 cells were seeded per well of a 96-well plate and infected with the shRNA-encoding AAV vectors at the indicated multiplicities of infection (MOI). Twenty-four hours later, AAV vector expressing Firefly and *Renilla* luciferases was added and incubated for 48 h. Luciferase assays were performed as described below. Mice of the FVB strain transgenic for the hAAT gene under a hepatocyte-specific promoter were reported (3). Animals were selected such that all groups had comparable average initial hAAT levels. AAV8 vectors were infused at doses of 3 × 10^11^or 6 × 10^11^ particles per mouse, in a total volume of 200 µl (using 1× PBS as diluent). Blood was collected at the indicated time points through retro-orbital bleeding, and plasma hAAT levels were determined via specific ELISA as described (3). All procedures were approved by the Animal Care Committee of the University Hospital of Cologne.

### Western blotting and immunofluorescence

To obtain samples for western blotting, 3 × 10^4^ cultured cells were homogenized in 200 µl of lysis buffer (2 mM EDTA, 100 mM Tris–HCl, pH 7.5, 4% sodium dodecyl sulphate, 20% glycerol, 10% ß-mercaptoethanol, 0.02% bromophenolblue). Before loading 20 µl of each sample on the gel, proteins were denatured by heating the sample for 5 min at 95°C. After the run, gels were blotted onto nitrocellulose membranes using a semi-dry blotting apparatus (Bio-Rad, Munich, Germany). To detect FLAG-Ago2 fusion proteins, a horseradish peroxidase-coupled anti-FLAG antibody (M2, F1804, SIGMA) was used at a 1:1000 dilution in TBST (Tris-buffered saline with 0.1% Tween-20), together with a peroxidase-conjugated anti-mouse secondary antibody (diluted 1:10000, #115-035-067, Jackson ImmunoResearch/Dianova, Hamburg, Germany) and an enhanced chemiluminescence system (Perkin Elmer, Rodgau, Germany). Human Ago2 was detected using aspecific rat antibody (#1149, kindly provided by Gunter Meister) diluted 1:50 in 5% bovine serum albumin, followed by Alexa Fluor 647-coupled secondary anti-rat antibody (diluted 1:500, Invitrogen). Mouse p53 was detected using the monoclonal 1C12 antibody (Cell Signaling Technology, Boston, MA, USA) and the abovementioned anti-mouse secondary antibody.

### cDNA and miRNA profiling

Total RNA for cDNA and miRNA profiling was isolated from cells in 10-cm dishes using 6 ml of TRIzol reagent (Applied Biosystems). Therefore, cells were incubated in TRIzol for 5 min, transferred into a 15-ml tube, supplied with 200 µl of chloroform per ml TRIzol, shaken vigorously and then centrifuged at 2885*g* for 30 min at room temperature. The aqueous phase was transferred to a fresh 15-ml tube, and total RNA was precipitated in isopropanol, washed with 75% ethanol and finally dissolved in 60 µl of RNase-free water. For cDNA profiling, Sentrix Human-6v3 Whole Genome Expression BeadChips (Sentrix Human WG-6; Illumina, San Diego, CA, USA) were used. To synthesize first and second strand cDNA and amplify biotinylated cRNA from the total RNA, an Illumina Totalprep RNA Amplification Kit was used according to the manufacturer's instructions. Hybridization to the BeadChip was also performed based on the manufacturer's instructions. A maximum of 10 µl of cRNA was mixed with 20 µl of GEX-HYB hybridization solution. The preheated 30 µl of assay sample was dispensed onto the large sample port of each array and incubated for 18 h at 58°C. Following hybridization, the samples were washed according to the protocol and scanned with a BeadArray Reader (Illumina). Raw data were exported from the Beadstudio software to R (http://cran.r-project.org/), and then quantile-normalized and log2-transformed. MiRNA expression profiling was performed using the miCHIP microarray platform as described (42,43). In brief, 500 ng of total RNA were labeled with a Cy3-conjugated RNA linker (Biospring, Frankfurt, Germany) and hybridized on the microarray. miCHIP is based on locked nucleic acid (LNA) technology, whereby LNA-modified Tm-normalized miRCURY capture probes (Exiqon, Denmark, miRBase v11) were printed on Codelink slides (GE Healthcare, Munich, Germany). Microarray images were generated using the Genepix 4200AL laser scanner (Molecular Devices, Biberach an der Riss, Germany) in batches using the Genepix auto PMT (Photo Multiplayer). The ‘MultiExperiment Viewer’ MeV was used to execute the statistical technique SAM (Significance Analysis of Microarrays) to identify dysregulated miRNAs in a set of microarray experiments with a 2-fold threshold.

### Quantitative real-time PCR

All quantitative real-time PCR (qRT-PCR) reactions were run on an ABI7500 thermo cycler (Applied Biosystems) using Power SYBR® Green PCR Master Mix (Applied Biosystems). All reactions were performed in triplicates in a 25-µl final volume. Primer sequences were CMVfor 5′ TGCCCAGTACATGACCTTATGG 3′, CMVrev 5′ GAAATCCCCGTGAGTCAAACC 3′, FASNfor 5′ AACTCCAAGGACACAGTCACCAT 3′, FASNrev 5′ CAGCTGCTCCACGAACTCAA 3′, PAK4for 5′ GGCAGGGTGAAGCTGTCAGA 3′, PAK4rev 5′ TCTCCGTCCACCATCTCAATC 3′. Myo10 was detected using a commercial primer (Qiagen, Quantitect primer assay QT00079296).

### Luciferase reporter assays

Transfection-based assays using *Renilla* or Firefly luciferase as RNAi targets were performed in cells grown in 96-well plates. Unless indicated otherwise in figure legends, the following plasmid amounts were used. In assays in which shRNA and Ago2 were expressed from two separate plasmids, each well was triple-transfected with 2.5 ng of psiCheck2 (Promega), 25 ng of shRNA and 37.5 ng of pCA2n plasmid. In cases where shRNA and Ago2 were instead co-expressed from a single construct, 25 ng of the respective plasmid were co-transfected with 10 ng of psiCheck2. Stuffer DNA (pBlueScriptIIS/K plasmid) was always added to 200 ng. In all cases, cells were harvested 48 h after transfection into lysis buffer supplied in the Dual-Glo luciferase kit according to the manufacturer’s protocol (Promega), and *Renilla* and Firefly luciferase activities were quantified on a GloMax96 microplate luminometer (Promega). Relative knockdowns were determined using the nontargeted luciferase and nonspecific control shRNAs as two references.

### Quantitation of mitotic indices

Parental or stably Ago2-expressing HeLaP4 cells were transfected with siRNAs and 30 h later fixed with 4% paraformaldehyde at room temperature for 30 min. Following 30 min permeabilization at room temperature using 0.2% Triton X-100, the cells were washed three times with 1× PBS and subsequently incubated for 30 min in 1× PBS and 5% fetal bovine serum (FBS) to block unspecific binding sites. This solution was then aspirated and the cells were incubated overnight at 4°C with a mouse monoclonal antibody against histone 3 phospho-serine 10 (PH-3, diluted 1:500 in 1× PBS with 5% FBS) (New England Biolabs, Frankfurt, Germany). Because PH-3 correlates with chromosome condensation during mitosis, it serves as a marker for dividing nuclei. The cells were washed three times with 1× PBS to remove unbound antibody and then stained with secondary Alexa Fluor 488-coupled anti-mouse IgG antibody (diluted 1:500, Invitrogen). To identify individual nuclei/cells, Hoechst fluorescent DNA binding dye was added. After a 2-h incubation at room temperature, the cells were again washed three times in 1× PBS and then left in wash buffer for subsequent automatic data acquisition. Fluorescence was measured in a Scan⁁Rscreening microscope (Olympus, Hamburg, Germany) using appropriate excitation and emission filters, and numbers of total nuclei and mitotic cells were recorded for each well. Finally, using voronoi-like watershed transformation and image analysis software kindly provided by Christoph Sommer (Swiss Federal Institute of Technology Zurich, Institute of Biochemistry), automated image segmentation was performed and the fraction of cells in mitosis (mitotic index) was calculated by dividing numbers of PH-3-positive cells by total nuclei counts.

### HBV assays

To measure activities of anti-HBV shRNAs, parental or stable Ago2-expressing Huh7 cells were co-transfected in a 24-well format with pTHBV2 together with sAg19 or sAg25 anti-HBV shRNAs, or luc29 as a control. In addition, they received a hAAT expression plasmid for later measurement of secreted hAAT protein for normalization purposes. Plasmid amounts per well were 230 ng of pTHBV2, 285 ng of phAAT and 285 ng of the shRNA construct. In all cases, cell supernatants were collected 48 and 96 h after treatment, and HBsAg was measured via standard Elisa (3).

### Vesicular stomatitis virus glycoprotein (VSV-G) assays

Parental or stable Ago2-expressing HeLaP4 cells were plated on 8-well μ-slides (Ibidi, Martinsried, Germany) at a density of 9 × 10^3^ cells per well and 1 day later transfected with siRNAs at a final 50 nM concentration. Medium was replaced after 4 h, and the cells were maintained for 38 h before they were transduced with an adenoviral vector encoding a temperature-sensitive Yfp-tagged mutant of vesicular stomatitis virus (VSV) glycoprotein (ts-O45-G) (44). Subsequently, the infected cells were incubated at 39.5°C and 5% CO_2_ for 6 h to allow ts-O45-G expression and accumulation in the endoplasmic reticulum (ER). Then, a synchronized release of ts-O45-G from the ER was induced by shifting the temperature to 32°C in the presence of 100 μg/ml cycloheximide. After a 1-h incubation, the cells were fixed with 3% paraformaldehyde for 20 min at room temperature, and ts-O45-G that had trafficked to the plasma membrane was immunostained with the mouse monoclonal antibody VG (recognizing the extracellular ts-O45-G epitope, generous gift from Kai Simons, MPI-CBG, Dresden, Germany) plus secondary goat-anti-mouse Cy5-conjugated antibody (GE Healthcare). In addition, cell nuclei were stained using 0.3 μg/ml Hoechst 33342. Images of total ts-O45-G, ts-O45-G on the plasma membrane and nuclei were acquired (36 positions per well) on an automated fluorescence microscope IX81 (Olympus) equipped with 10× UplanSApo objective (NA 0.4). Image quality was inspected visually, and image analysis was then performed using Scan⁁R Analysis software (Olympus). Next, cells expressing too much ts-O45-G (∼7.5% of the brightest cells) or not transduced with the adenoviral vector (∼5% of the dimmest cells) were removed from the analysis. For each siRNA, 10 000–12 000 cells were further analyzed. The ts-O45-G secretion rate in a single cell was calculated as ratio of plasma membrane-incorporated ts-O45-G to the total amount of expressed protein. A median of secretion rates in all cells was then deduced based on all acquired images in each well. R and the ‘RNAither' package (http://www.bioconductor.org/) were used for normalization of experimental replicates. For cross-experiment comparisons, the median of the negative control (‘All Stars’ siRNA) was subtracted from each measurement, and the variance was adjusted by dividing by the standard deviation of the negative control. Consequently, negative values indicate inhibition of ts-O45-G secretion, using a threshold of −1.5 for hit scoring.

### HCV assays

All siRNAs were prediluted to 0.5 µM and spotted in 10 µl of aliquots (triplicates per siRNA) into 96-well plates. Per well, 0.75 µl of HiPerfect (Qiagen) and 50 µl of Opti-MEM® I (Invitrogen/Gibco) were then added and incubated with the siRNAs for 10 min at room temperature. Subsequently, 7 × 10^3^ Huh7 cells (parental or stably Ago2-transfected) were plated in each well in a 150-µl volume and left overnight at 37°C and 5% CO_2_. Next morning, media were replaced and the cells were incubated for 2 days for gene silencing to occur. They were then infected with *Renilla* luciferase-expressing HCV [JcR2a (45)] at a multiplicity of infection of 0.5. After 24 h, the cells were washed with 100 µl of 1× PBS, resuspended in 30 µl of lysis buffer (1% Triton X-100, 25 mM glycylglycine, 15 mM MgSO_4_, 4 mM ethylene glycol tetraacetic acid (EGTA), 1 mM dithiothreitol (DTT), pH 7.8) and stored at −70°C for later determination of luciferase activities. Therefore, 50 μl of luciferase assay buffer (25 mM glycylglycine, 15 mM MgSO_4_, 4 mM EGTA, 15 mM K_2_PO_4_, pH 7.8) supplemented with 3.6 ng/ml of Coelenterazin were added, before luciferase activity was measured for 1 s per well at 485 nm in a plate luminometer (Mithras LB 940; Berthold Technologies, Freiburg, Germany).

### Rift Valley fever virus assays

Lentivirally transduced A549-Ago2 cells were seeded at 7 × 10^3^cells per well into 96-well plates and transfected with 10 µM siRNA using HiPerfect (Qiagen) according to the manufacturer’s protocol (siMAVS: 5′ CCCACAGGGUCAGUUGUAUTT 3′; negative control: Qiagen AllStars Negative Control). After a 48-h incubation, cells were infected with Rift Valley fever virus (RVFV) clone 13, carrying the *Renilla* luciferase gene in place of the NSs (nonstructural protein on the S segment) coding region (kind gift from Friedemann Weber, Marburg). Forty-eight hours after infection, cells were lysed and luciferase activity was assessed as described above for HCV.

### Kinase RNAi screen

A sub-library of the genome-wide siRNA library siGENOME (Dharmacon, Thermo) targeting the human kinome was used for the kinase RNAi screen (46). The library contained 779 siRNA pools, each consisting of four synthetic siRNA duplexes (dissolved in RNase-free water). Before RNAi screening, the siRNA pools as well as additional positive (PLK1, UBC) and negative (RLUC) siRNA controls with known phenotypes were distributed into black 384-well multititer plates (BD Falcon, Becton Dickinson GmbH, Heidelberg, Germany) and stored at −20°C until the experiment. The gene UBC encodes a polyubiquitin precursor protein associated with ubiquitination. The gene PLK1 encodes a serine/threonine-protein kinase that performs several important functions throughout M phase of the cell cycle. Both genes cause cell death when silenced by RNAi and were thus used as technical positive siRNA transfection controls. The gene RLUC encodes for *Renilla* luciferase, which is absent in mammalian cells. Its silencing was used as negative control, as RLUC siRNA was expected to lack phenotypic effect. The siRNA library was arrayed in multititer plates using a Biomek FX200 liquid handling system (Beckman Coulter, Krefeld, Germany). Each well contained 5 µl of a 200-nM pool of four synthetic siRNA duplexes. Library siRNAs were spotted in columns 5–24, while the remaining columns were used for controls. In a preparatory step before RNAi screening, predistributed and frozen siRNA pools were thawed at room temperature for 30 min. Reverse transfection of cells with siRNA pools was performed by delivering 15 µl of Roswell Park Memorial Institute medium (RPMI) (Invitrogen) containing 0.05 µl of Dharmafect1 (Dharmacon). After 30 min incubation at room temperature, 1 × 10^3^ cells in 30 µl of DMEM medium with 10% FBS were added to the siRNA transfection mix. Both control and Ago2-overexpressing HeLaP4 cell lines were reverse-transfected in two replicates. Plates were incubated for 72 h at 37°C and 5% CO_2_. The experiment was terminated by paraformaldehyde fixation (45 min at room temperature) and subsequent immunostaining (1 h at room temperature) using α-tubulin (FITC-labeled, diluted 1:500, F2168, SIGMA) and α-actin (TRITC-phalloidin, diluted 1:3000, P1951, SIGMA) antibodies as well as Hoechst 33 342 DNA stain (diluted 1:8000, SIGMA). All dispensing steps were performed with a Multidrop Combi dispensing system (Thermo). The morphology of the treated cells was observed using a BD Pathway 855 fluorescence microscope (20× objective).

For data analysis, generated image files were processed using the EBImage package (47) as described (48). After image segmentation, each identified cell was characterized by a set of 46 morphological descriptors (Supplementary Table S2) that were computed on different channels (red: actin, green: tubulin, blue: DNA). The characteristics of all cells within each well of a 384-well multititer plate were then averaged in a vector summarizing all 46 numerical descriptors. Next, the Kolmogorov-Smirnov statistics between sample and negative control (RLUC) wells was calculated, resulting in a numeric vector profiling each siRNA treatment against the control condition. To assess the similarity of RNAi-induced phenotypes in the control and Ago2-overexpressing HeLaP4 cell line, the Euclidean distance between two vectors of the same siRNA treatment was calculated and defined as the phenotypic distance. The latter was finally used for identification of siRNAs with more pronounced phenotypes in the Ago2-overexpressing line as compared with control cells.

### HIV assay

A collection of 460 different commercially available siRNAs (Ambion, Applied Biosystems) targeting 230 genes was reverse-transfected into HIV-permissive HeLaP4 cells (wild-type or overexpressing Ago2) through an established reverse transfection protocol (49). The individual genes were silenced for 36 h before infection with a Gfp-expressing HIV-1 NL4-3 derivative [AGFP (50)]. Another 36 h later, cells were fixed with 4% paraformaldehyde and stained with Hoechst 33258 before image acquisition in a fully automated epifluorescence Scan^R screening microscope equipped with the Scan^R acquisition software (Olympus). Automated image segmentation was performed to determine green fluorescence protein (GFP) mean intensities and to classify infected and noninfected cells. To reveal enhancing or inhibiting effects on viral infection/replication, the GFP expression data were statistically analyzed in R, using cellHTS and the Bioconductor package RNAither (51).The normalization per plate was based on a negative control (NS#1) also from Applied Biosystems. The entire workflow was published recently (52).

## RESULTS

**Figure 1.**
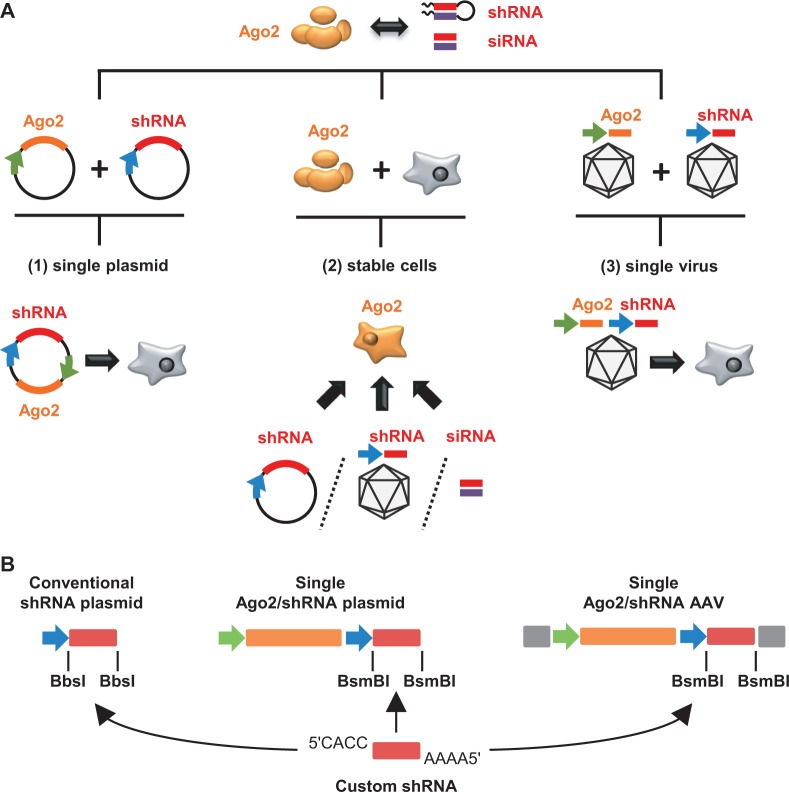
Three strategies to enhance si or shRNA efficiencies in mammalian cells (schemes). (**A**) Indicated on top is the strict si/shRNA dependency on Ago2 (drawn with its four major domains) for potent target mRNA cleavage. Depicted underneath are previous proofs-of-concept that intracellular Ago2 levels determine RNAi efficiency. Shown at the bottom are the three improved and broadly applicable strategies implemented here to overcome the rate-limiting nature of endogenous Ago2 and to increase RNAi potency: (1) Co-expression of shRNA together with Ago2 from a single plasmid, engineered to permit rapid and simple shRNA cloning; (2) stable Ago2 cDNA integration into cell lines that can be transfected with siRNAs or shRNA plasmids, or transduced with shRNA-encoding viral vectors; or (3) shRNA and Ago2 co-expression*in cis* from a viral vector as alternative to transient plasmid transfection and to improve multiple aspects of *in vivo* RNAi. (**B**) Illustration of the compatibility and versatility of our different shRNA expression plasmids. As indicated by the arrows, an shRNA composed of two annealed oligonucleotides and carrying the indicated overhangs can be cloned into any of our constructs, either for mere shRNA expression (left), or for co-expression with Ago2 from a plasmid (center) or an Adeno-associated viral (AAV) vector (right, gray boxes symbolize AAV packaging signals). See panel A for other symbols/colors and Supplementary Figure S1 for more details.

Our aim in this work was to devise broadly applicable and user-friendly strategies to enhance si or shRNA activities via transient or stable Ago2 overexpression. Toward this goal, we engineered a set of plasmids and viral vectors to permit custom shRNA co-expression together with a codon-optimized human Ago2 cDNA. In addition, we derived and characterized clonal cell lines stably overexpressing this Ago2 variant based on three cell types that are routinely and frequently used in RNAi experiments—HeLa(P4), 293T and Huh7(.5). An overview over these three avenues is shown in Figure 1A.

### Bi-cistronic Ago2/shRNA expression plasmids yield increased shRNA activities

Our first aim was to create a plasmid construct that would boost RNAi without a need to co-deliver Ago2 *in trans* and that would concurrently permit convenient shRNA cloning. Accordingly, we merged a cistron expressing a codon-optimized human Ago2 cDNA from a CMV promoter with a second cassette for shRNA expression. The latter contains a human U6 promoter followed by two unique restriction sites for rapid and directed shRNA cloning as annealed oligonucleotides (3) (Figure 1B, see ‘Materials and Methods’ section and Supplementary Figure S1 for more details).

**Figure 2.**
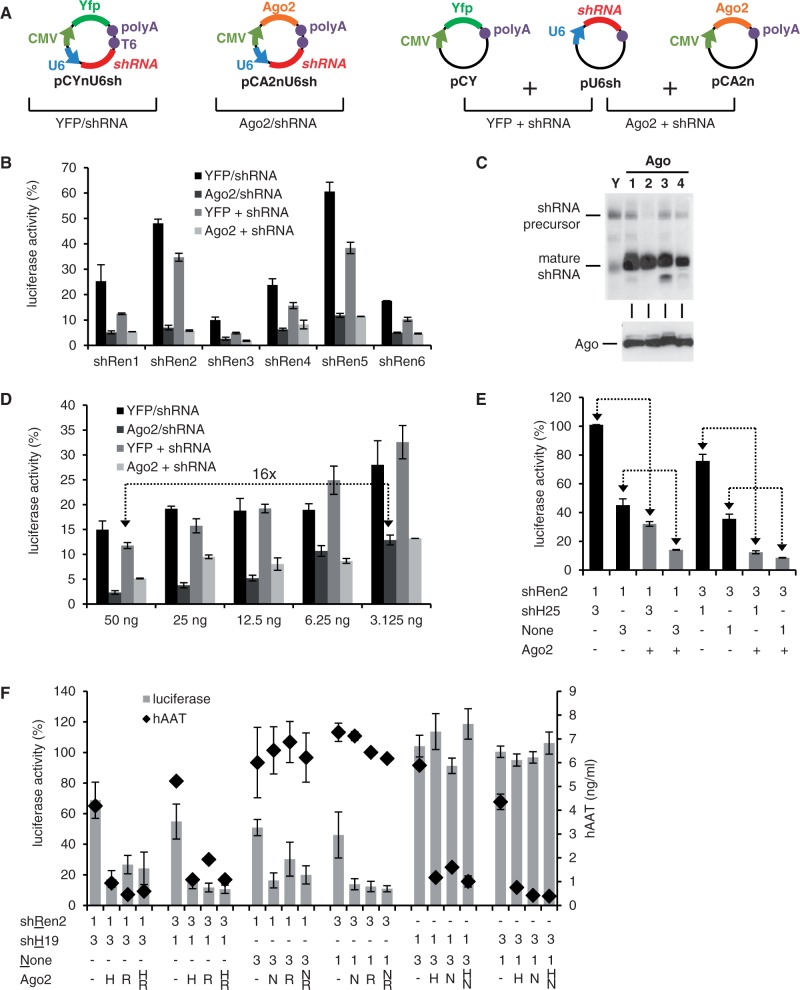
Improvement of shRNA knockdowns by transient Ago2 co-expression *in cis*. (**A**) Scheme depicting the principal constructs and experimental settings. (**B**) HeLaP4 cells were co-transfected with psiCheck2 and plasmids encoding six different shRNAs against *Renilla* luciferase. In addition, Ago2 or Yfp were co-expressed *in cis* or *in trans* (see panel A for all combinations). *Renilla* luciferase activity was normalized to Firefly luciferase in each sample, and then to that of a nonsilencing control shRNA. (**C**) Top: Northern blot after shRen3 co-transfection into 293T cells with each of the four human Ago proteins (lane numbers denote Ago1–4) or Yfp (Y) as control. Bottom: Western blot confirming equal Ago protein expression. Note that while all four proteins stabilize mature shRNA strand steady-state levels, only Ago2 moreover improves shRNA precursor processing (evidenced by a reduction of the respective signal in the northern blot). (**D–F**) Luciferase assays in HeLaP4 cells to study shRNA dose responses and competition. (D) Dose–response analysis in cells transfected with the indicated decreasing amounts of shRNA plasmids. See panel A for data analysis. The arrow symbolizes that Ago2 co-expression permits the use of 16-fold lower shRNA plasmid amounts to achieve the same knockdown as without exogenous Ago2. (E) Combinatorial RNAi experiment in which cells were co-transfected with two plasmids, one encoding an shRNA against *Renilla* luciferase (shRen2), and the other an irrelevant shRNA (shH25) or none at all. Numbers indicate molar ratios of these two plasmids. In addition, all cells received the psiCheck2 reporter. At the bottom of the legends, Ago2 co-expression from the shRen2 plasmid is indicated by ‘+’, while ‘−’ is the corresponding Yfp controls. The arrows highlight sample pairs with or without Ago2 co-expression to illustrate how Ago2 concurrently improves the efficiency of both shRNAs and relieves their competition. (F) Combinatorial RNAi assay akin to panel E, except that targets for both shRNAs were included (*Renilla* luciferase and hAAT). Numbers below the graph are molar plasmid ratios, and letters designate the plasmid that encoded Ago2 (see underlined letters on the left). All bars in this figure represent means ± SD (*n* = 3), except for panel F, where they are means ± SEM (*n* = 6).

We next inserted six different shRNAs (one per construct) against *Renilla* luciferase into this bi-cistronic plasmid and compared knockdown efficiencies in transfected cells to controls co-expressing Yfp instead of Ago2. As additional controls, we individually expressed shRNA and either Ago2 or Yfp from two separate constructs (Figure 2A). All six shRNAs were markedly enhanced by Ago2 co-expression *in cis* or *in trans* by at least 2- and up to 10-fold, yielding >90% knockdown efficiencies in all cases (Figure 2B and Supplementary Figure S2). The increased shRNA performance correlated with a stronger signal for the mature shRNA guide strand in small RNA northern blots in the presence of Ago overexpression (Figure 2C), confirming shRNA stabilization as one of the mechanisms underlying Ago2-dependent RNAi improvements (27).

Using a moderately potent shRNA as example, we next demonstrated that the Ago2 effect is stable for plasmid doses spanning over one order of magnitude, persistently producing knockdown efficiencies between 87 and 98% (Figure 2D). Notably, even the lowest tested amount of the bi-cistronic Ago2/shRNA construct already gave a robust knockdown, which matched that obtained with a 16-fold higher dose of the standard shRNA expression plasmid (Figure 2D, arrows). Especially at the higher doses, the improvements in shRNA efficiency were more pronounced with the single *cis* construct than with separate shRNA and Ago2 expression. This may reflect another advantage of the dual plasmid, namely, a more uniform co-delivery of shRNA and Ago2 to the same cell.

An interesting question was whether increased Ago2 levels would also relieve competition between two co-delivered shRNAs, an adverse effect that frequently hampers RNAi experiments. We thus co-transfected cells with different ratios of plasmids encoding shRNAs against either luciferase or human hAAT, or with a control lacking shRNA. We used a weak anti-luciferase shRNA (shRen2, Figure 2B) in combination with our most potent anti-hAAT shRNA (3), to effectively out-compete luciferase knockdown and to facilitate detection of benefits from Ago2 expression. Indeed, the anti-hAAT shRNA diminished the efficiency of the anti-luciferase shRNA in a dose-dependent manner, culminating in a complete block of luciferase knockdown at a 3-fold excess of the anti-hAAT shRNA (Figure 2E, leftmost bar). Notably, Ago2 co-expression from the anti-luciferase shRNA construct largely alleviated this competition and improved luciferase knockdown by 3- to 6-fold (arrows in Figure 2E). At the 3:1 anti-luciferase:hAAT shRNA ratio, the block from the anti-hAAT shRNA was completely overcome by expression of ectopic Ago2, as evidenced by comparable luciferase knockdowns in the presence or absence of competitor shRNA (Figure 2E, two gray bars on the right).

To test whether Ago2 co-expression would also concomitantly enhance knockdown of the two shRNA-associated targets, we repeated the co-transfections with plasmids encoding *Renilla* luciferase or hAAT. To avoid bias toward one of the two RNAi triggers, the 25mer anti-hAAT shRNA was replaced with a weaker 19mer. As shown in Figure 2F, Ago2 expression indeed substantially enhanced knockdown of the two targets. Importantly, Ago2 delivery from one plasmid already sufficed for this effect, and Ago2 co-delivery from both constructs provided no additional boost. Together, these results illustrate the usefulness of our strategy for combinatorial RNAi studies as well as its broad compatibility with preexisting shRNA constructs.

### Ago2 co-expression also enhances Dicer-independent shRNAs

**Figure 3.**
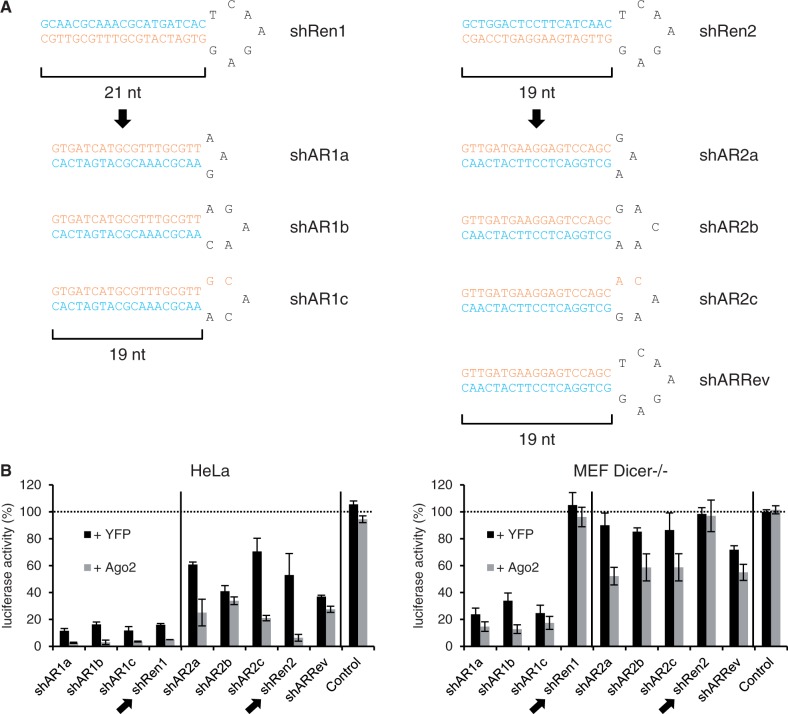
Ago2 overexpression increases knockdown by minimal shRNAs even in the absence of Dicer. (**A**) Scheme depicting the two original shRNAs (top, shRen1/2) and seven derivatives designed according to the rules by Liu *et al.* (33). Antisense strands are in orange, sense strands in blue. All nine shRNAs were inserted into our new dual plasmid backbone co-expressing either Ago2 or Yfp *in cis*. (**B**) Analysis of the nine shRNAs in Dicer-competent (HeLa) or -deficient (MEF Dicer−/−) cells. Shown are luciferase assays after co-transfection of these plasmids together with psiCheck2. All values were normalized to Firefly luciferase and to the empty shRNA plasmid. ‘Control’ is an unrelated shRNA against hAAT. The two conventional shRNAs are highlighted by arrows, while the dotted line depicts the 100% mark (no knockdown). Bars represent means ± SD (*n* = 3).

The shRNAs used above had a traditional design, i.e. 19–25mer stems, placement of the antisense strand at the 3′ arm and a 7mer loop sequence. Interestingly, several groups have recently reported novel small RNA duplexes that circumvent Dicer cleavage and favor direct Ago2 processing. We were particularly intrigued by findings by Liu *et al.* that reduction of the stem to 17–19 nt and of the loop to 3–6 nt can render an shRNA a Dicer-independent Ago2 substrate (termed ‘AgoshRNA’) and can concurrently shift the bias of RISC incorporation to the 5′ strand (33). We accordingly hypothesized that this Ago2-dependent shRNA blueprint should represent a superior template for our Ago2 co-expression strategy especially in cells that lack functional Dicer, and that may thus resist conventional shRNAs. To validate this idea, we re-designed two of our anti-luciferase shRNAs following the rules by Liu and colleagues (Figure 3A) and evaluated the resulting constructs in Dicer-expressing HeLa cells versus Dicer-deficient murine embryonic fibroblasts (MEFs). In HeLa cells, both the conventional shRNAs as well the cognate AgoshRNAs mediated target knockdown that was enhanced by additional Ago2 expression (Figure 3B, left). In striking contrast, the two standard shRNAs were inactive in the cells lacking Dicer, whereas the seven AgoshRNA duplexes continued to markedly inhibit luciferase (Figure 3B, right). Even more notably, all Dicer-independent shRNAs were further enhanced by Ago2 overexpression in the Dicer-deficient MEF, resulting in up to 90% silencing (versus up to 75% without Ago2 co-delivery). Conversely, neither of the conventional shRNAs could be boosted by ectopic Ago2, confirming their strict dependency on Dicer processing and hence complete inertness in Dicer-deficient cells regardless of Ago2 levels.

### Stable Ago2 overexpression in human cell lines improves RNAi efficiency

**Figure 4.**
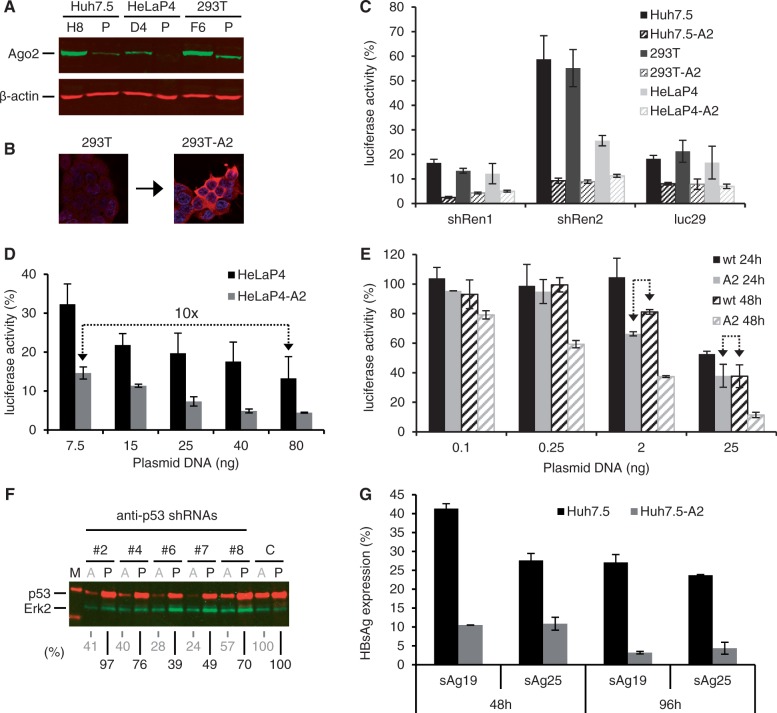
Enhancement of shRNA efficiencies by stable Ago2 overexpression in human cell lines. (**A**) Western blot illustrating Ago2 overexpression in our three selected lead candidates (H8, D4, F6) derived from the indicated human cell lines (P, parental). β-actin served as loading control. (**B**) Representative Ago2-specific immunofluorescence further exemplifying increased Ago2 levels in stably transfected cells. Red, Ago2; blue, DNA (Hoechst stain). (**C**) Validation of improved RNAi efficiencies in stable Ago2-expressing cell lines (suffix ‘-A2’) via transfection with plasmids encoding different anti-luciferase shRNAs and psiCheck2. *Renilla* (targeted by shRen1/2 shRNAs) or Firefly (targeted by luc29 shRNA) luciferase were knocked down in three parental cells lines (filled bars) or the corresponding Ago-2-overexpressing lead derivatives (dashed bars). All luciferase reads were normalized to those after transfection of a control shRNA against hAAT. (**D**) Titration series in which wild-type or Ago2-overexpressing HeLaP4 cells were co-transfected with psiCheck2 and the indicated amounts of shRNA2-encoding plasmid. Values were normalized as in (C). The arrow highlights that stable Ago2 expression permits to use 10-fold lower shRNA plasmid doses to achieve the same knockdown as in the parental cells. (**E**) Kinetics study in which wild-type (wt) or Ago2-overexpressing (A2) cells were transfected with psiCheck2 and the indicated amounts of shRNA2-encoding plasmid. *Renilla* luciferase knockdown was quantified 24 or 48 h later. Values were normalized as in (C). The arrows highlight that cell lines overexpressing Ago2 mediate substantially accelerated RNAi at a given shRNA dose. (**F**) Western blot against p53 after transfection of Ago2-overexpressing (A) versus parental (P) cells with five different anti-p53 shRNAs (numbers #2–8 above the blot). Erk2 served as loading control. C, control shRNA (luc29); M, protein marker. Numbers below the blot indicate percent remaining p53 protein compared with the corresponding control (set to 100). (**G**) Knockdown of HBV surface antigen (HBsAg) in parental (black) or Ago2-expressing (gray) Huh7.5 cells 48 or 96 h after transfection with pTHBV2 and two different anti-HBsAg shRNAs. Shown are remaining HBsAg levels in the cell supernatant, normalized to an irrelevant anti-hAAT shRNA. The improvements in shRNA potency in the Ago2 cells ranged from 2-fold (sAg25, 48 h) to up to 10-fold (sAg19, 96 h). All bars in this figure represent means ± SD (*n* = 3).

In a prior screen for rate-limiting RNAi factors, we had observed that stable Ago2 overexpression in human Huh7 liver cells resulted in increased RNAi activity (17). To validate whether this concept is broadly useful to boost RNAi efficiencies, we comprehensively analyzed the effects of stable Ago2 overexpression in human cell lines that are broadly used in RNAi experiments: HeLaP4 (a HeLa derivative overexpressing primary and secondary HIV-1 receptors), 293T (human embryonic kidney cells) and Huh7.5 (an Huh7 subclone with increased permissiveness for HCV). In all three cases, we obtained at least 20% positive clones (HeLaP4: 22%, Huh7.5: 64%, 293T: 82%), which stably and robustly (13- to 25-fold) overexpressed the codon-optimized Ago2 cDNA after transfection and selection (Figure 4A and B).

For characterization of these cells, a first important question was whether elevated Ago2 expression caused inadvertent effects, considering the key role of Ago2/RISC for cellular physiology. Indeed, we observed a broad spectrum of growth behaviors among the stable Ago2 clones (exemplified for HeLaP4 in Supplementary Figure S3A). None of the Ago2 overexpressing clonal cell lines grew faster than the cognate original cell line, which is notable regarding controversies about the role of Ago2 in cellular transformation (see Discussion). For all cell lines, we succeeded in isolating several clones that displayed growth kinetics identical or highly similar to the parental cells (e.g. D4 or E12 in Supplementary Figure S3A, respectively). These clones also exhibited indistinguishable morphologies (not shown), further supporting the absence of adverse Ago2 effects. In addition, miRNA and cDNA profiling revealed only negligible changes over the parental cells, proving that Ago2 overexpression has not essentially affected endogenous small RNA or gene expression in the clonal lines (Supplementary Figure S3B and Supplementary Table S1). For all subsequent studies, we chose the clonal cell line (e.g. D4 for HeLaP4, Supplementary Figure S3A) that best preserved the characteristics of the original cells.

To determine whether the elevated Ago2 levels translated into improved RNAi, we first tested the same anti-luciferase shRNAs as in the transient transfections before. A similar, up to 10-fold enhancement of RNAi activity was observed with all shRNAs (again including the inherently weak shRen2) in the Ago2 lines as compared with the parental cells (Figure 4C). When assessing the shRNA dose response, we found that stable Ago2 expression permitted the use of at least 10-fold lower plasmid amounts to achieve the same knockdown as in parental cells (Figure 4D). Even at the highest plasmid dose, the Ago2-overexpressing cells still yielded 3-fold better RNAi activity. In addition to dose, we compared knockdown kinetics at two different time points after shRNA transfection of stable Ago2 versus parental cells. At each plasmid dose, silencing efficiency in the Ago2-overexpressing cells at 24 h matched or surpassed that achieved at 48 h in the parental cells (Figure 4E, arrows), illustrating the capacity of stably Ago2-expressing cell lines to not only yield more potent, but also faster RNAi. The superior RNAi potency was further validated with independent shRNA sets against p53 (Figure 4F) and HBV (Figure 4G). Moreover, stable Ago2 cells concurrently improved the potency of two co-delivered shRNAs to silence their respective targets (Supplementary Figure S3C), exemplifying the usefulness of these cells for combinatorial RNAi (compare also Figure 2E and F). Additional ectopic Ago2 expression by transient transfection of Ago2 cell lines yielded only marginal or no further improvements, indicating that Ago2 levels are no longer rate-limiting in these stable cells (Supplementary Figure S3C and D).

### Ago2-overexpressing cell lines yield better siRNA potency and improved RNAi phenotypes

Our next aim was to assess whether stable Ago2 cell lines would also benefit RNAi applications other than shRNA transfection, especially siRNA experiments for which transient Ago2 co-expression from a plasmid would be impractical. We therefore tested siRNA-mediated knockdown of several individual cellular genes in the new cell lines and additionally performed a comparative siRNA screen. In all assays, particular focus was put not only on target gene inhibition, but also on the resulting RNAi phenotype.

**Figure 5.**
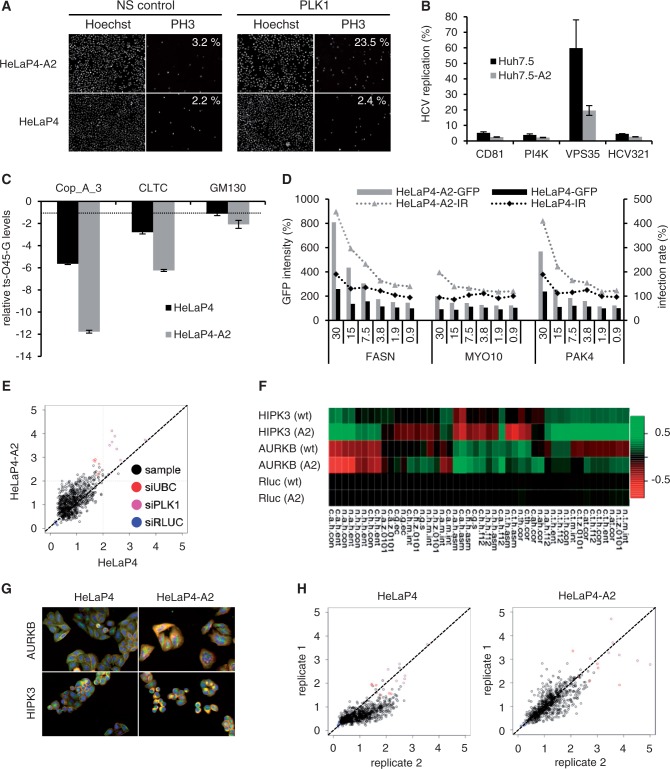
Stable Ago2 overexpression improves siRNA potencies and associated phenotypes. (**A**) Visualization and quantification of mitotic cells (phenotypic readout, see ‘Materials and Methods’ section for calculation) after transfection of siRNAs against *PLK1* into Ago2-expressing or parental HeLaP4 cells. Note the 10-fold increase in the Ago2 clone (upper rightmost panel). NS, nonsilencing. (**B** and **C**) Further examples for more pronounced phenotypes in stably Ago2-transfected cells (gray bars) with siRNAs against HCV host factors (B) or genes encoding proteins involved in vesicle transport (C). See ‘Materials and Methods’ section for assay details. Plotted in (C) are ratios of ts-O45-G protein detected at the plasma membrane versus total protein. The dotted line in (C) denotes the assay threshold (−1.5). Bars are means ± SEM (*n* = 4). (**D**) Dose–response analysis of three siRNAs (numbers indicate transfected amounts in nM) against the indicated HIV-1 host restriction factors. Intensity of Gfp expression (encoded by the reporter HIV strain) and infection rates (IR) served as phenotypic readouts. Measured values were normalized to a negative control siRNA. Shown are representative results. Note how stable Ago2 overexpression improved both Gfp expression and infection rates. For assay details, see ‘Materials and Methods’ section and Börner *et al.* (52). (**E**) Comparison of phenotype strengths in an siRNA screen against the human kinome in wild-type versus Ago2-overexpressing HeLaP4 cells. X and Y axes are the Euclidean distance (z-score normalized) of individual samples to the negative control in the two cell lines. The diagonal line represents samples showing equal magnitude of phenotypic effects in both cell lines, while points above the diagonal line are samples with stronger phenotypes in the Ago2 cells. That the vast majority of samples with significant phenotypes (z-score >2) and the positive controls distribute above the diagonal line exemplifies that phenotypes are stronger in the Ago2 cell line. See ‘Materials and Methods’ section for the control siRNAs. (**F**) Examples of target genes (rows) and parameters (columns, see Supplementary Table S2 for abbreviations) documenting improved knockdowns and phenotypes in Ago2-expressing (A2) as compared with wild-type (wt) cells. Values and colors represent Kolmogorov-Smirnov statistics: red means that a sample feature is larger than the control feature, green means the opposite and black indicates equal features. AURKB, Aurora kinase B; HIPK3, Homeodomain-interacting protein kinase 3. (**G**) Further documentation of enhanced phenotypes in the Ago2 cells. Shown are two representative siRNA targets (same as in F) with greater than 2-fold phenotypic distances in the Ago2 cell line. Colors represent actin (red), tubulin (green) and DNA/nuclei (blue). (**H**) Illustration of the higher RNAi assay reproducibility in the Ago2 cells as compared with the parental line (shown for two independent replicates each).

As a first candidate, *PLK1*—a regulator of human chromosome segregation and cell division—was silenced using a specific siRNA, and the ensuing phenotype was analyzed by determining mitotic indices and nuclei counts. The *PLK1* siRNA yielded a marked increase in mitotic cells in the Ago2-overexpressing HeLaP4 as compared with the parental cell line (Figure 5A). Similarly, siRNA-mediated knockdown of *INCENP* (inner centromer protein, another key regulator of chromosome segregation) resulted in a 3-fold higher reduction of mitotic indices in the Ago2 clone (data not shown). We observed equivalent RNAi improvements for siRNAs against human host factors for three different viruses, HCV, VSV or HIV. In the case of HCV, four siRNAs against cellular factors required for viral entry or replication yielded a stronger phenotype (HCV inhibition) in the Ago2-overexpressing clone as compared with parental Huh7.5 (Figure 5B). Robust Ago2-dependent augmentation of RNAi phenotypes was also observed in HeLaP4-Ago2 cells after transfection with siRNAs against three cellular proteins involved in secretory membrane trafficking (Figure 5C). Using a temperature-sensitive mutant (ts-O45-G) of the VSV-G glycoprotein as a model cargo protein and its transport from the ER to the plasma membrane as readout, we found an ∼2-fold boost of all three siRNA phenotypes (i.e. an inhibition of membrane trafficking, reflected by lower ratios of fluorescent ts-O45-G protein at the plasma membrane versus total protein; gray bars in Figure 5C). Remarkably, for the siRNA against GM130 (a peripheral protein of the Golgi complex), the phenotype only manifested in the Ago2 clone where the ts-O45-G crossed the assay threshold of −1.5 (see ‘Materials and Methods’ section for details).

Next, we tested siRNAs against 117 putative host factors for HIV-1 that were recently identified using an automated high-throughput siRNA screening platform (52) (Börner *et al.*, manuscript in preparation). Notably, 50 of these initial hits could be validated in the HeLaP4-Ago2 cells, while only 24 scored positive in parental HeLaP4 (hit criteria were at least three independent siRNAs displaying the same phenotype in the validation screen as in the primary screen). Three representative candidates were then studied regarding their siRNA dose response, using mean GFP expression from a reporter HIV strain and infection rates as two different readouts. As shown in Figure 5D, stable Ago2 expression markedly improved both phenotypes for all three HIV host restriction factors and over a wide siRNA dose range.

These consistent results led us to test the HeLaP4-Ago2 cells in a high-throughput, multi-parametric siRNA screen whose phenotypic readout was the viability or fitness of cells (46). We therefore transfected parental HeLaP4 and the stable Ago2 derivative with an siRNA library targeting ∼800 genes implied in the human kinome (four siRNAs per gene), and subsequently microscopically analyzed the cells for 46 individual parameters (Supplementary Table S2). Two observations were remarkable: First, the majority of siRNA phenotypes were markedly enhanced in the Ago2 clone (Figure 5E, points above the diagonal line), with 36 of the 779 siRNA pools (4.6%) showing a greater than 2-fold distance as compared with the correspondingly transfected control cells. Notably, siRNA efficiencies were increased for 296 of the 779 screened genes (38%), i.e. the siRNAs targeting these genes reached the hit criteria exclusively in the presence of Ago2 overexpression and concurrently yielded a more consistent phenotype when compared with the parental cells. Representative examples of target genes whose knockdowns and associated phenotypes were clearly enhanced in the Ago2 cell line are shown in Figure 5F, G. Second, the Pearson correlation coefficient between two independent replicates was higher in the Ago2 cells than in the parental line (0.76 versus 0.7, Figure 5H). Together, these results imply that stably overexpressing Ago2 in human cells not only improves siRNA phenotypes, but also enhances reproducibility between experiments, both of which are essential advantages in particular for high-throughput RNAi analyses.

Finally, we assessed the possibility that stable Ago2 overexpression could alleviate unintended dysregulation of cellular gene expression by reducing off-targeting effects. To this end, Ago2-expressing and parental HeLaP4 cells were transfected with the siRNA against Myo10 (Figure 5D) and then analyzed by cDNA profiling. We indeed found a 42% reduction in the overall number of altered genes (wild-type cells: 460, Ago2 clone: 268) as well as a trend to overall milder dysregulations in the Ago2 cell line (Supplementary Figure S4).

### Rapid and potent generation of Ago2 cell lines through lentiviral transduction

The studies above had all been conducted in clonal cell lines that were selected and expanded after Ago2 plasmid transfection. The success of this strategy depends on several parameters, especially the amenability of the starting cells to DNA transfection, as well as the efficiency and duration of selection. Because these parameters can be limiting for some cell types, we aimed to implement an alternative means for rapid and potent generation of polyclonal Ago2-overexpressing cell pools.

To this end, we constructed a recombinant VSV-G-pseudotyped lentivirus expressing the codon-optimized Ago2 cDNA together with a blasticidin resistance cassette, and then evaluated its potential to stably transduce a panel of human cell lines: 293T and A549 (adherent cells) as well as SupT1 (a nonadherent hard-to-transfect T-cell line). Already within 2 weeks after transduction, cell pools stably overexpressing Ago2 to an extent comparable with the stable clones generated through transfection were obtained for all three lines (Supplementary Figure S5A and B). These lentivirus-derived cell pools behaved identical to the above described Ago2-overexpressing cell lines and likewise yielded 2- to 10-fold enhanced RNAi in functional assays with anti-luciferase shRNAs (Supplementary Figure S5C and D). The capacity of these cell pools for improved RNAi was further analyzed using an siRNA against mitochondrial antiviral signaling protein (*MAVS*), a critical gene in the interferon induction pathway, and RVFV replication as phenotypic readout. Accordingly, wild-type or Ago2-overexpressing A549 cells were transfected with *MAVS*-specific or control siRNA and cultured for 48 h to allow silencing to occur. Subsequently, the cells were infected with two different doses of *Renilla* luciferase-encoding RVFV. As expected, knockdown of *MAVS* (and thereby of the interferon response) rescued viral replication. Notably, this rescue was substantially larger in the A549-Ago2 cells, doubling the readout window for this assay (Supplementary Figure S5E).

### Ago2/shRNA co-expression from Adeno-associated viral vectors improves *in vitro* RNAi

Our final aim was to devise robust strategies that would translate the benefits of Ago2 overexpression into *in vivo* RNAi applications. However, neither the plasmids nor the lentiviral reagents seemed ideal for this purpose, owing to concerns about low *in vivo* efficiencies and specificities as well as potential safety issues. In contrast, none of these reservations apply to recombinant vectors derived from AAV. While the AAV2 prototype is already highly efficient in many cells, we and others have further expanded the breadth of AAV target cells *in vitro* and *in vivo* through the use of other natural viral serotypes or synthetically engineered capsids (53,54). Additional advantages of AAV vectors over other viral systems include their apathogenicity, their rare genomic integration (alleviating worries about genotoxicity) and their amenability to large-scale production and purification.

We therefore engineered an AAV vector genome to co-encode the codon-optimized Ago2 cDNA (or Yfp as control) together with an empty U6 cassette for versatile shRNA cloning. Subsequently, an anti-luciferase shRNA was inserted into the Ago2- or Yfp-expressing AAV vector plasmids, and the resulting constructs were packaged into viral particles. For these vectors, conventional AAV plasmids were used, which generate ss DNA genomes for packaging into, and delivery by, the viral capsids. In parallel, the same shRNA was cloned into a double-stranded (ds) vector genome, i.e. a derivative that carries two inverted copies of the transgene and hence mediates faster and more robust gene expression in certain cells (55). Notably, ds vectors only accommodate ∼2.2 kb of foreign DNA (half the size of a ss vector) and thus cannot be engineered to co-express shRNAs with Ago2 (whose cDNA is 2.6 kb long).

**Figure 6.**
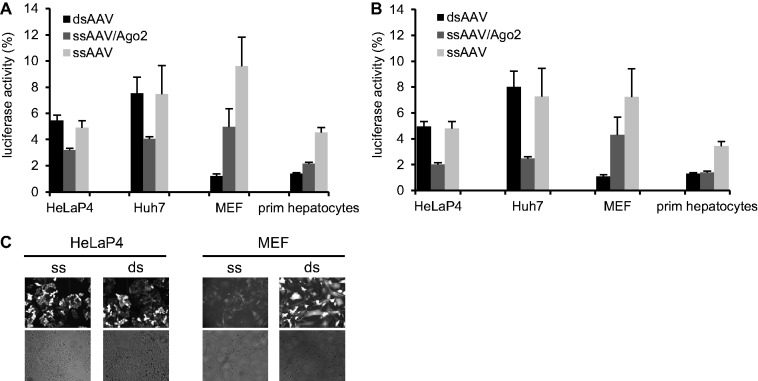
Ago2/shRNA co-expression from AAV vectors can improve RNAi in different mammalian cell types. (**A** and **B**) The indicated four cell types were infected with three different AAV vectors (expressing Ren3 shRNA) at MOIs (i.e. particles per cell) of 2.5 × 10^5^ (A) or 5 × 10^5^ (B). In addition, the cells were infected with an AAV vector encoding the same dual luciferase expression cassette used in all transfection-based experiments. Values were normalized to those with a control AAV expressing an irrelevant anti-hAAT shRNA. prim, primary. (**C**) The superior performance of the Ago2/shRNA-co-encoding ssAAV vector in HeLaP4 and Huh7 cells (medium gray bars in A and B) correlated well with the high infectability of these cells (depicted for the high MOI group and HeLaP4 as examples in C). Yfp/Gfp co-encoded on the vectors (the ssAAV variant co-expressed Yfp) is shown on top, corresponding transmission pictures are underneath. In contrast, primary cells (MEFs and primary hepatocytes) were only potently transduced by the dsAAV vector (A–C). Bars are means ± SD (*n* = 3).

We next compared these three shRNA vectors—ssAgo2/shRNA, ssYfp/shRNA, dsGfp/shRNA—in four different cell types, including primary MEFs and hepatocytes, which are notoriously difficult to transfect. In line with all the previous plasmid data, Ago2 co-expression *in cis* from the ss vector improved shRNA activity by ∼2-fold as compared with the Yfp/shRNA-expressing ss control (Figure 6A and B; light versus medium gray bars). Nonetheless, the dsAAV vector outperformed both ss variants in the primary cells, with the exception of the high AAV dose in the hepatocytes at which both ds and ssAgo2 vector performed equally well (Figure 6B). The higher knockdown efficiency from the ds vector correlated with the stronger reporter gene expression in these two cell types, confirming ss to ds DNA conversion as a rate-limiting parameter for AAV transduction in particular cells (Figure 6C).

Notably, the other two cell lines (HeLaP4, Huh7) are highly susceptible to ssAAV infection and hence independent of this conversion step. In these cells, we thus observed no differences between the ssYfp and dsGfp vectors lacking Ago2 (Figure 6A–C). Remarkably, the Ago2/shRNA-encoding ssAAV was the best performing vector in these highly infectable cells and gave a 2-fold better knockdown than the other two variants. This confirms that Ago2 is the predominant rate-limiting factor if DNA conversion is not restricting, which in turn implies that the bi-cistronic Ago2/shRNA AAV vector may be useful in a large variety of cell types.

### AAV-Ago2/shRNA vectors mediate stronger and safer RNAi in adult murine livers

**Figure 7.**
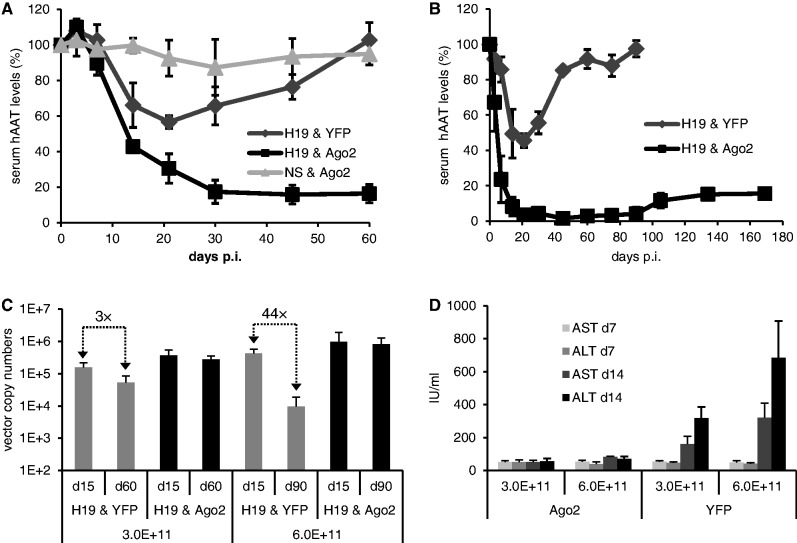
Ago2 co-expression from shRNA-encoding AAV vectors mediates stronger, longer and safer *in vivo* RNAi. (**A** and **B**) hAAT-transgenic mice were peripherally infused with 3 × 10^11^ (A) or 6 × 10^11^ (B) AAV particles per animal. The vectors encoded the shown shRNA and Ago2 or Yfp combinations (H19, hAAT19 shRNA; NS, nonsilencing control luc29). p.i., postinfection. Serum hAAT values were normalized to those before vector injection (set to 100%). (**C**) qRT-PCR-based quantification of AAV vector numbers in livers from the mice in A, B at different time points. Numbers at the bottom are the injected particle doses. Arrows indicate drops in vector copy numbers over time in animals lacking additional Ago2. The high vector persistence in the Ago2 cohorts is indirect proof that Ago2-co-expression had alleviated *in vivo* shRNA toxicity. (**D**) Analysis of serum markers of liver damage in the animals from A and B. AST, aspartate transaminase; ALT, alanine transaminase. The substantial dose-dependent increase in the Yfp cohorts (right half) indicates liver damage in the absence of additional Ago2 co-expression. The fact that serum markers remained unaltered in the Ago2 cohort (left half) implies Ago2-induced alleviation of shRNA hepatotoxicity. All values in this figure are means ± SD. The number of animals per group was *n* = 6 until day 15 p.i., and *n* = 3 afterward.

To investigate whether AAV-mediated Ago2/shRNA co-expression *in cis* can also boost RNAi *in vivo*, we exploited transgenic mice encoding hAAT. This model had also been used when we previously discovered cytotoxicities from Ago2 saturation (3,17). In these earlier studies, AAV doses >1 × 10^11^ particles per mouse had caused detectable hepatotoxicities, in turn resulting in vector losses due to liver damage and hepatocyte repopulation, and in drops in RNAi efficiencies. To test whether these adverse effects could be overcome by Ago2 co-expression, we deliberately infused a 3-fold higher dose (3 × 10^11^ vector particles per mouse) of the AAV co-encoding Ago2 and anti-hAAT shRNA, and then compared serum hAAT levels with mice injected with a Yfp/shRNA control. Already 1 week after particle administration, mice transduced with the Ago2-co-encoding vector displayed a more robust hAAT knockdown, which increased until a stable efficiency of ∼80% (Figure 7A). In stark contrast, the Yfp/shRNA vector did not achieve >40% knockdown, and silencing was only transient as hAAT levels returned to normal over the next 1.5 months, reminiscent of our prior observations related to hepatotoxicity (3).

To test whether Ago2 co-expression could alleviate toxicities from even higher shRNA doses, we infused a new cohort of hAAT-transgenic mice with 6 × 10^11^ particles per animal. For the Yfp/shRNA vector, this resulted in an increased knockdown efficiency of up to 60% at 3 weeks after injection. However, hAAT levels then again gradually returned to normal, indicative of liver toxicity (Figure 7B). Conversely, the Ago2/shRNA vector yielded even stronger and accelerated RNAi as compared with the 3 × 10^11^ dose, culminating in >80% hAAT knockdown already at day 14 (versus day 30 for the lower vector dose, Figure 7A). This robust knockdown persisted until the end of the experiment (5.5 months), except for a minor decline starting after 3 months. Notably, the stable >85% knockdown exceeds the best *in vivo* RNAi efficiencies with this particular shRNA cassette that we had observed in all our prior experiments (3,17).

As indicated, a likely explanation for the transient nature of the vector lacking additional Ago2 expression was loss of transduced hepatocytes, and thus vector genomes, due to toxic saturation of the endogenous RNAi machinery. To confirm this hypothesis, we measured AAV vector copy numbers in total liver DNA from the various mouse cohorts. Indeed, we noted a substantial dose-dependent drop at late time points in animals treated with the Yfp/shRNA vector, as opposed to the high vector copy numbers that persisted in the Ago2/shRNA groups (Figure 7C). As additional proof, we detected an AAV dose-dependent elevation of two serum markers for liver damage in the mice infused with the Yfp/shRNA vector at day 14. On the contrary, the samples from the animals that had received the Ago2 co-expressing vector remained at baseline levels, even at the highest dose of 6 × 10^11^ AAVs per mouse (Figure 7D). This suggests that Ago2 co-expression not only increases shRNA efficiency *in vivo*, but that the elevated Ago2 levels also buffer potential adverse effects over a wide shRNA dose range, both of which could be important features for future clinical RNAi applications.

## DISCUSSION

The present work was fueled by persistent concerns within the RNAi field that even after a decade of intensive research, the efficiency, specificity or safety of this technology still do not truly match the high anticipations. Our study essentially complements a long list of prior efforts to improve RNAi and to foster its *in vitro* and *in vivo* application, which segregate into two categories: The first are strategies that aim at enhancing the RNAi effector itself, e.g. by increasing siRNA stability or shRNA strand specificity, or by using miRNAs as scaffolds for RNAi expression [for reviews, see e.g. (2,15,56)]. The second category is devised to act on a global level, by improving the cellular capacity to process and use ectopic RNAi triggers. A promising example is the small molecule enoxacin, which can boost si/shRNAs in cells and mice, with only minor effects on cellular gene or miRNA expression (57–59).

Our own three strategies fall into this second category, as they consistently aimed at improving RNAi knockdowns without a need to re-design available si or shRNAs. Their common denominator is overexpression of human Ago2, a central and rate-limiting component of the RNAi pathway whose saturation restricts si/shRNA efficiency, hampers combinatorial knockdowns and contributes to cytotoxicity (11,16,17). For robust Ago2 overexpression, we either encoded a codon-optimized cDNA on the same plasmid or viral vector as the shRNA, or stably expressed Ago2 within cultured cells. As demonstrated, these three methodological avenues can enhance the potency of all conventional RNAi triggers—transfected si or shRNAs or transduced shRNAs—in mammalian cells *ex* or *in vivo*. While previous reports had already indicated that ectopic Ago2 expression can enhance si or shRNA activity (16,17,60,61), the present work represents an important advance in our understanding of Ago2/RNAi biology and its implications for RNAi applications, for three reasons: First, the comprehensive and systematic evaluation of the benefits of Ago2 overexpression in cell lines, primary cells and animals underscores the enormous potential to improve a wide range of typical small- and large-scale RNAi experiments, including high-content screens. Second, the alleviation of shRNA-induced *in vivo* hepatotoxicities with Ago2-co-encoding vectors reaffirms prior models that a major hurdle for therapeutic RNAi is saturation of the cellular pathway with ecoptic triggers, and concurrently provides a new solution. Third, the customizable sets of new plasmids, cells and viruses reported here should assist in the implementation, optimization and routine application of Ago2 overexpression strategies in other labs.

The predominant and most readily detectable manifestation of the values of Ago2 overexpression is the increased potency of si- or shRNA-mediated knockdowns. Importantly, all three approaches reported here are compatible with preexisting RNAi triggers and should hence provide this benefit for any si or shRNA. To validate this feature, we tested a variety of si and shRNAs that were already present in the lab or newly designed based on specific rules. We consistently observed 2- to 10-fold Ago2-dependent enhancements of their activities with numerous targets and cell lines, and regardless of whether Ago2 was co-expressed from a plasmid, stably integrated into cells or co-delivered *in vitro* or *in vivo* by a viral vector. This suggests that up to one order of magnitude represents the typical range of RNAi improvement that one can expect from this strategy, without concern for si/shRNA or target sequence selectivity. It moreover implies that Ago2 elevation will also benefit experiments involving collections of RNAi triggers with varying efficiencies, including special si/shRNA designs. Indeed, we confirmed the power of Ago2 co-expression for a new class of shRNAs that function without Dicer and have an inherent strand bias toward the 5′ strand (33). This hints that bi-cistronic Ago2/AgoshRNA plasmids will also be useful in cells in which Dicer is mutated, depleted or inhibited, a phenomenon that is increasingly found in human cancers or other diseases including viral infections (62–66). Moreover, the AgoshRNA strand bias indicates that their use in combination with Ago2 overexpression could be a promising future strategy to reduce relative off-target activity from the passenger shRNA strand, when the latter is deliberately placed at the 3′ end of these shRNAs.

Next to RNAi potency, a second important parameter that we addressed is safety, an aspect with particular relevance for future translational applications. This parameter comprises two scenarios: (i) concerns about cellular perturbations originating from Ago2 overexpression *per se*, and, vice versa, (ii) Ago2-induced alleviation of toxic saturation effects caused by ectopic RNAi triggers. Noteworthy in the first context are several recent studies that reported controversial outcomes of Ago2 overexpression (deliberately induced or naturally occurring) in different cell lines and tumors. These included inhibition or enhancement of cell proliferation, migration and tumorigenic properties (67–69), occasionally accompanied by altered miRNA or mRNA expression (68,70). Still, others observed no adverse changes at all on stable Ago2 overexpression in various cells (71,72), supporting the use of such cells as tools to study fundamental Ago/RNAi biology (73). While our studies were not designed to conclusively resolve the role of Ago2 as oncogene or tumor-suppressor, we also noted a spectrum of cellular phenotypes among our initial Ago2 cell lines. Remarkably, none of our clonal lines or cell pools exhibited increased proliferation or other signs of enhanced transformation. Moreover, the absence of a clear correlation between growth retardation and Ago2 expression levels hints that the cellular defects were rather owing to detrimental Ago2 cDNA integration or other particular clonal properties. As with any stable cell line, it is thus advisable to judiciously characterize lead candidates before further use. As shown here, preselection by analyses of growth kinetics can help in the isolation of useful Ago2 cell lines, as it repeatedly allowed us to derive clonal lines that robustly and stably overexpress Ago2 while exhibiting wild-type proliferation, morphology and miRNA and cDNA transcriptomes. This generally implies that vital endogenous Ago2 functions are not necessarily rate-limiting unless cellular RNAi homeostasis is disturbed by exogenous RNAi triggers (see next paragraph), explaining why Ago2 upregulation as such may be unproblematic. Once established, Ago2 overexpression can persist for long periods, as our cells continued to express high Ago2 levels, and to grow and look normally, even after 6 months in culture. Importantly, Ago2 overexpression is also well tolerated *in vivo*, as there were no signs of toxicity when we expressed Ago2 in murine livers from a separate AAV vector (17), or from the novel bi-cistronic AAV in the present study. This is in line with our additional observations in the previous study that Ago2 overexpression did not affect the activity of endogenous miRNAs toward a cognate imperfect target, further supporting that Ago2 expression *per se* poses no safety concern. Congruently, two other recent studies likewise reported that Ago2 overexpression did not perturb *Xenopus* embryonic development or CNS function in tadpoles (60,74).

As noted, a second side of safety as a key parameter in Ago2 overexpression strategies are the benefits in the presence of ectopic RNAi triggers, i.e. under conditions where endogenous Ago2 levels can become rate-limiting. The results in the present work are consistent with the model that Ago2 plays an exclusive and fragile role within the mammalian RNAi pathway, and that its titration by excessive levels of artificial RNAi triggers can be detrimental, especially *in vivo* (4). Current evidence are the increased hepatic RNAi persistence and reduced serum markers for liver damage in mice receiving both shRNAs and Ago2, verifying that *in vivo* Ago2 overexpression can alleviate cytotoxicity from shRNA over-abundance. This reinforces the idea that Ago2 overexpression counteracts shRNA-mediated saturation effects, by reinstating a critical mass of active RISC for use by the ectopic and the endogenous RNAi molecules. Modeling of the relative amounts of the various RNAi components in mammalian cells, notions of shRNA toxicities in other organs and species, as well as a comprehensive meta-analysis of adverse effects in previous siRNA screens all further support a dose-dependent RNAi competition/saturation model (13,75). These studies [reviewed in detail elsewhere (4)] and our new data unanimously draw the picture that mammalian RNAi mechanisms and especially Ago2 functions are deliberately balanced in an intact cell and prone to exogenous perturbation. In the future, it will be interesting and informative to more thoroughly investigate whether these dose-dependent Ago2 saturation effects coincide with dysregulated miRNA expression and/or functionality, considering the aforementioned recent findings of Ago2-dependent processing of individual miRNAs, such as miR-451(26,28–30). In fact, we previously reported that hepatic shRNA overexpression perturbs processing of the liver-specific miR-122 (3). Our new Ago2-encoding plasmids and vectors should be useful to study whether the alleviation of cytotoxicity by Ago2 overexpression involves normalization of disturbed miRNA parameters, and to investigate what other Ago2-associated mechanisms may underlie and thwart ectopic RNAi saturation. Indeed, work from Diederichs and colleagues as well as our own data imply that Ago2 overexpression can induce further beneficial effects, including increases in mature shRNA abundance, stability and specificity (16,26,27), which are all factors that may additionally enhance RNAi potency and safety.

The versatile strategies and tools reported here may find wide applications in the RNAi community as they promise to improve a variety of experiments, from single or combinatorial knockdowns in cells, to long-term silencing in animals. The selection of a specific approach—Ago2 co-expression from plasmids, vectors or cell lines—will depend on the application. To enhance existing shRNA constructs in cell culture, one option is to re-clone the cassettes or shRNAs into the customizable Ago2 plasmid. Alternatively, one could exploit one of the Ago2 cell lines (if suitable) or generate new clones, using our plasmids or lentiviral vector for stable Ago2 transfection or transduction, respectively. From our experiences, we do not expect major difficulties provided the resulting clones are carefully monitored for adverse effects. Moreover, the same lines can be used to boost individual siRNAs or entire RNAi libraries. The latter option should hold multiple benefits for large-scale RNAi screening experiments: First, that more si/shRNAs will be rendered functional should increase the number of hits in primary screens and facilitate their secondary validation. Second, the higher si/shRNA activity will permit to reduce effective doses and replicate numbers, which will in turn minimize assay costs and time. Third, the relief from competition for Ago2 will improve combinatorial knockdowns, e.g. to better suppress mutating viral targets (10), or to simultaneously dissect multiple cellular pathways. Fourth, the preference of Ago2 for perfect RNAi duplexes and the use of minimal si/shRNA doses should reduce off-targeting and hence improve the stringency of phenotypic readouts, as indeed suggested by our observation of reduced global gene dysregulation in siRNA-transfected Ago2 cells. Still, it must be pointed out that our Ago2 overexpression strategies are primarily aimed at improving the on-target efficiency of RNAi triggers, rather than relieving their off-target potential. Data obtained in stable Ago2 cell lines, or with Ago2-expressing plasmids or vectors, thus warrant the same scrutiny and independent experimental validation as those acquired in conventional RNAi assays.

Lastly, future *in vivo* RNAi applications should benefit from the new Ago2/shRNA-co-expressing AAV vectors, which merge higher RNAi efficiencies with lower toxicities. It will be interesting to compare this strategy with alternative vector designs that likewise aimed at improving shRNA potency and alleviating toxicity, such as tissue-specific promoters or shRNA embedding in miRNA scaffolds (76,77). Two unique advantages of the Ago2 approach are that it is compatible with, and likely able to boost, any custom RNAi expression cassette, and that it should broaden the window of mature siRNA doses that are tolerated by the target cell. The latter is important considering that *in vivo* shRNA doses may fluctuate owing to experimental variations in AAV production, titration or application, or owing to inherent shRNA parameters that can affect the levels of fully processed Ago2 substrates (3). The dual Ago2/shRNA vectors are moreover superior to a coinfection strategy using two separate AAVs (17), as they require fewer costs, labor and time for virus production. It should finally be rewarding to juxtapose our new vector design with advanced AAV technology, which permits virus re-targeting to numerous nonhepatic tissues (53), with the latest expression cassettes for combinatorial RNAi (10,78,79), as well as with the AgoshRNA design, which may provide an increase in strand specificity (see above) also *in vivo*. Collectively, these outlooks fuel optimism that the strategies and reagents presented here will bring the community one step closer to fully unlocking the potential of RNAi as a surrogate genetic tool for gene annotation and as a promising clinical modality in humans.

## SUPPLEMENTARY DATA

Supplementary Data are available at NAR Online.

## FUNDING

Cluster of Excellence CellNetworks [EXC 81]; CHS-Foundation; VIROQUANT project, which was funded by the German Federal Ministry of Education and Research [BMBF, 0313923]. Funding for open access charge: Cluster of Excellence CellNetworks.

*Conflict of interest statement*. None declared.

## Supplementary Material

Supplementary Data
