# Supplementary material for: Robust RNAi enhancement via human Argonaute-2 overexpression from plasmids, viral vectors and cell lines
Source: Nucleic Acids Res. 2013 Sep 17;41(21):e199. doi: 10.1093/nar/gkt836 (PMC3834839; doi:10.1093/nar/gkt836)
Supplement: Supplementary Data [file supp_41_21_e199__index.html]

Robust RNAi enhancement via human Argonaute-2 overexpression from plasmids, viral vectors and cell lines — Robust RNAi enhancement via human Argonaute-2 overexpression from plasmids, viral vectors and cell lines — Supplementary Data 

# Robust RNAi enhancement via human Argonaute-2 overexpression from plasmids, viral vectors and cell lines

## Supplementary Data

files

**Files in this Data Supplement:**

- Supplementary Data - pdf file
