# Supplementary material for: Robust RNAi enhancement via human Argonaute-2 overexpression from plasmids, viral vectors and cell lines
Source: Nucleic Acids Res. 2013 Sep 17;41(21):e199. doi: 10.1093/nar/gkt836 (PMC3834839; doi:10.1093/nar/gkt836)
Supplement: Supplementary Data [file supp_gkt836_nar-02142-met-g-2013-File009.pdf]

Supplementary Material for

Börner *et al.*

Robust RNAi enhancement via human Argonaute-2 over-expression from plasmids, viral vectors and cell lines

CONTENTS:

- **Supplementary Figures 1-5**
- **Legends for Supplementary Figures 1-5**
- **Supplementary Tables 1-2**

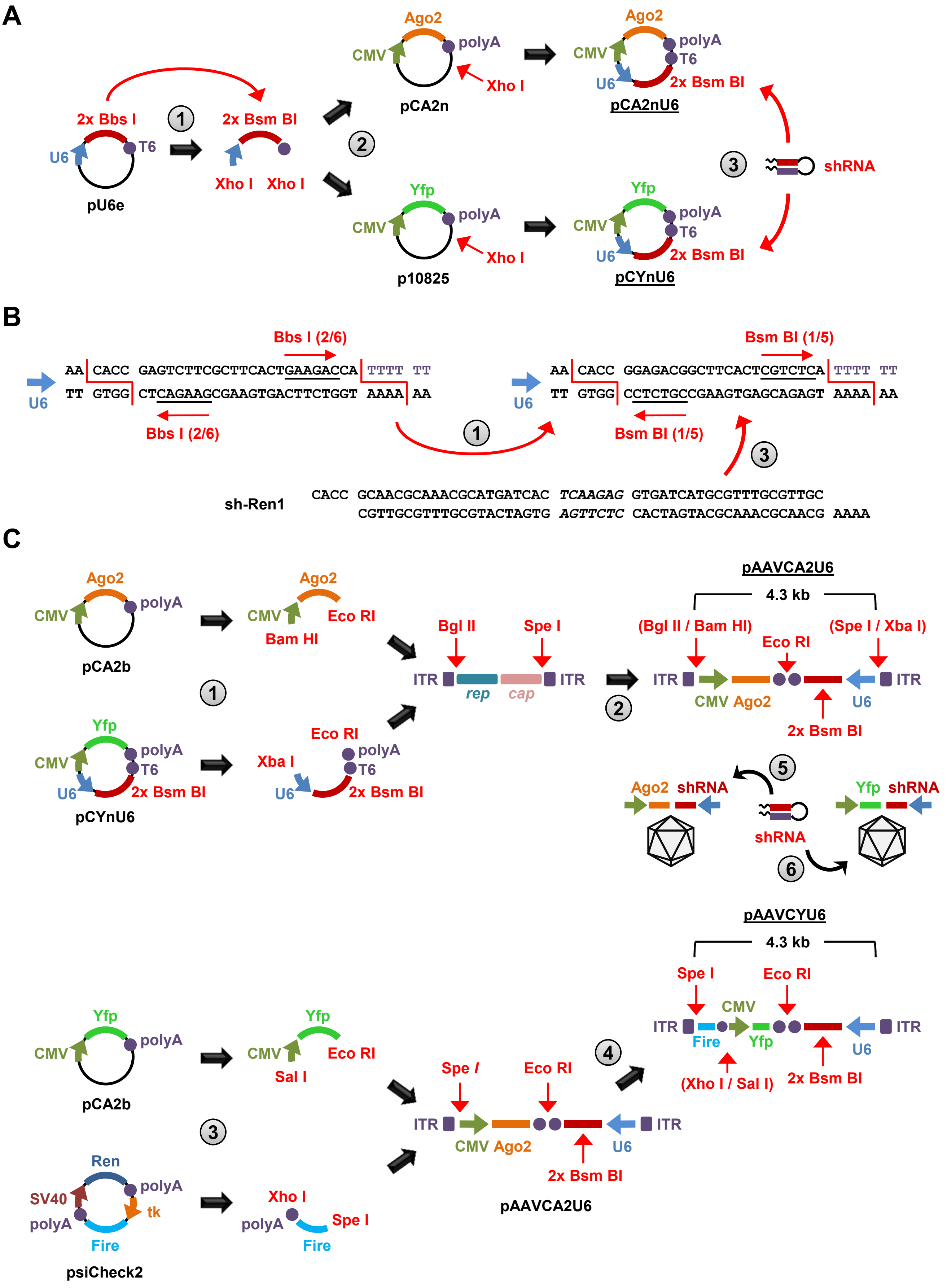

Börner *et al*. Supp Fig 1

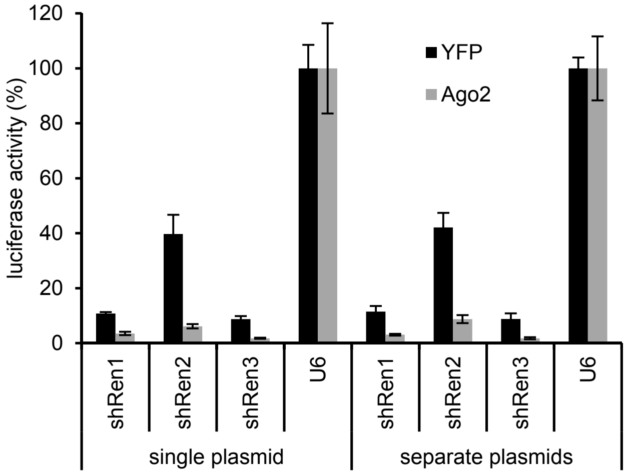

Börner *et al*. Supp Fig 2

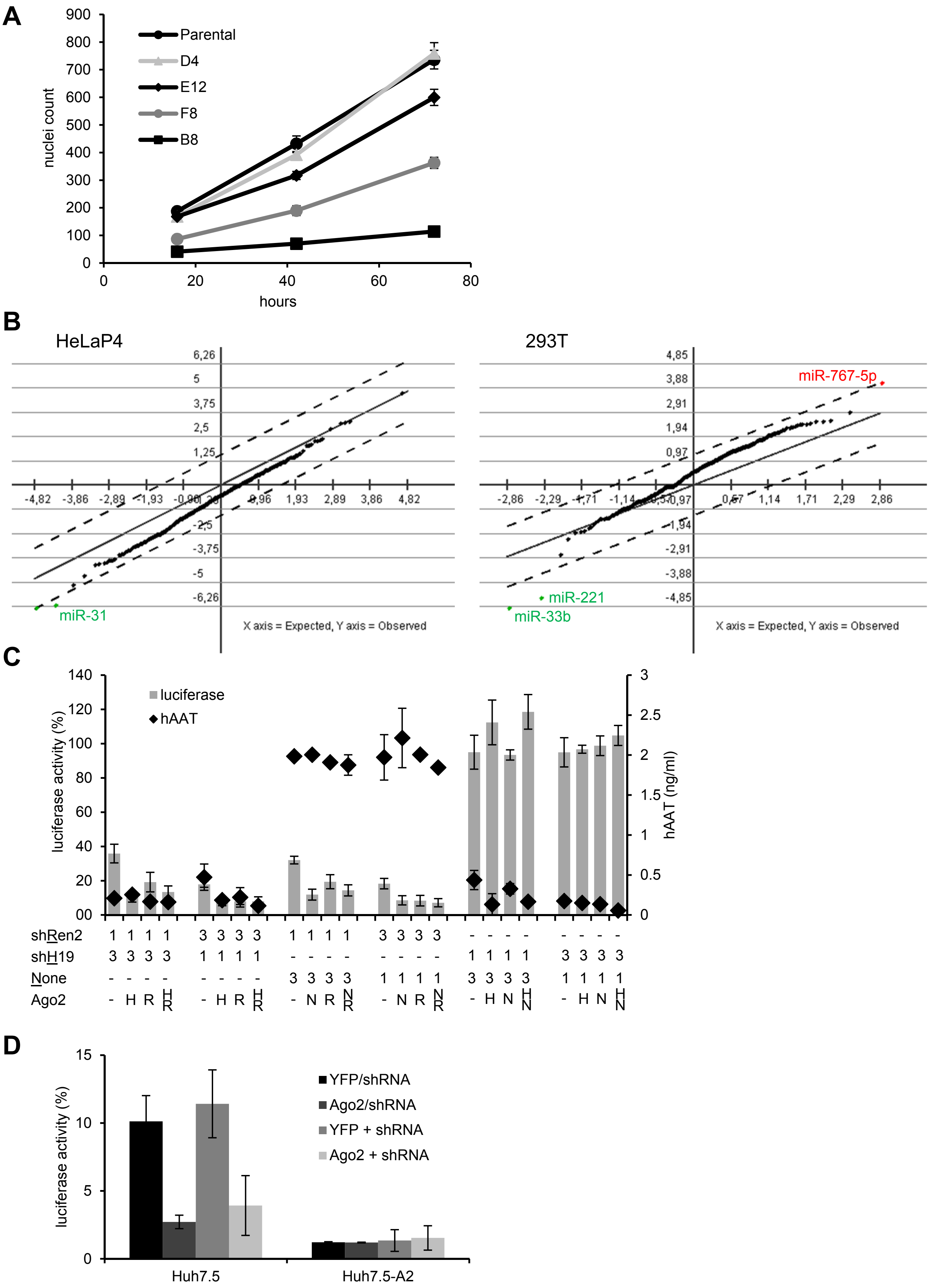

Börner *et al*. Supp Fig 3

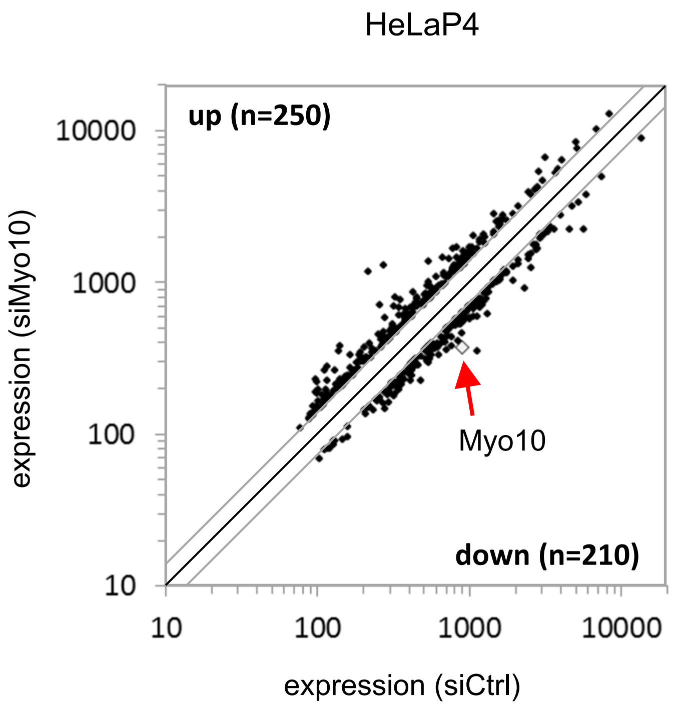

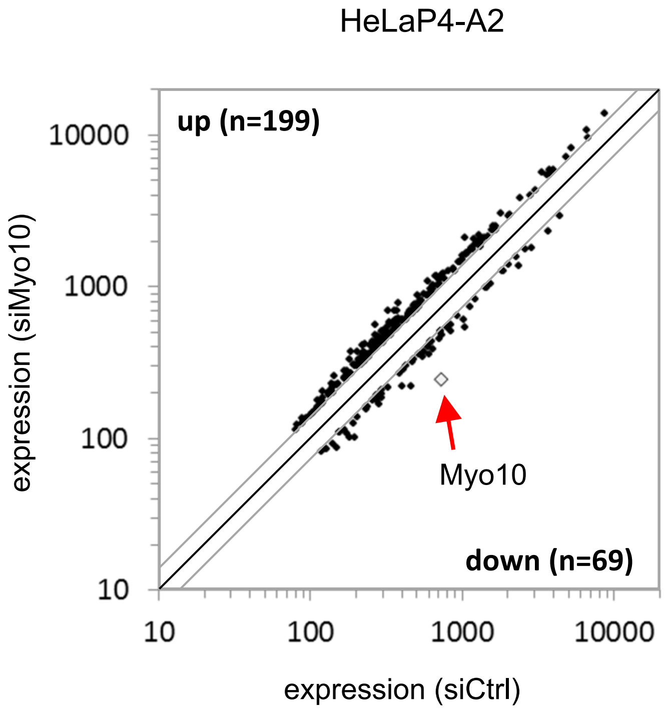

Börner *et al*. Supp Fig 4

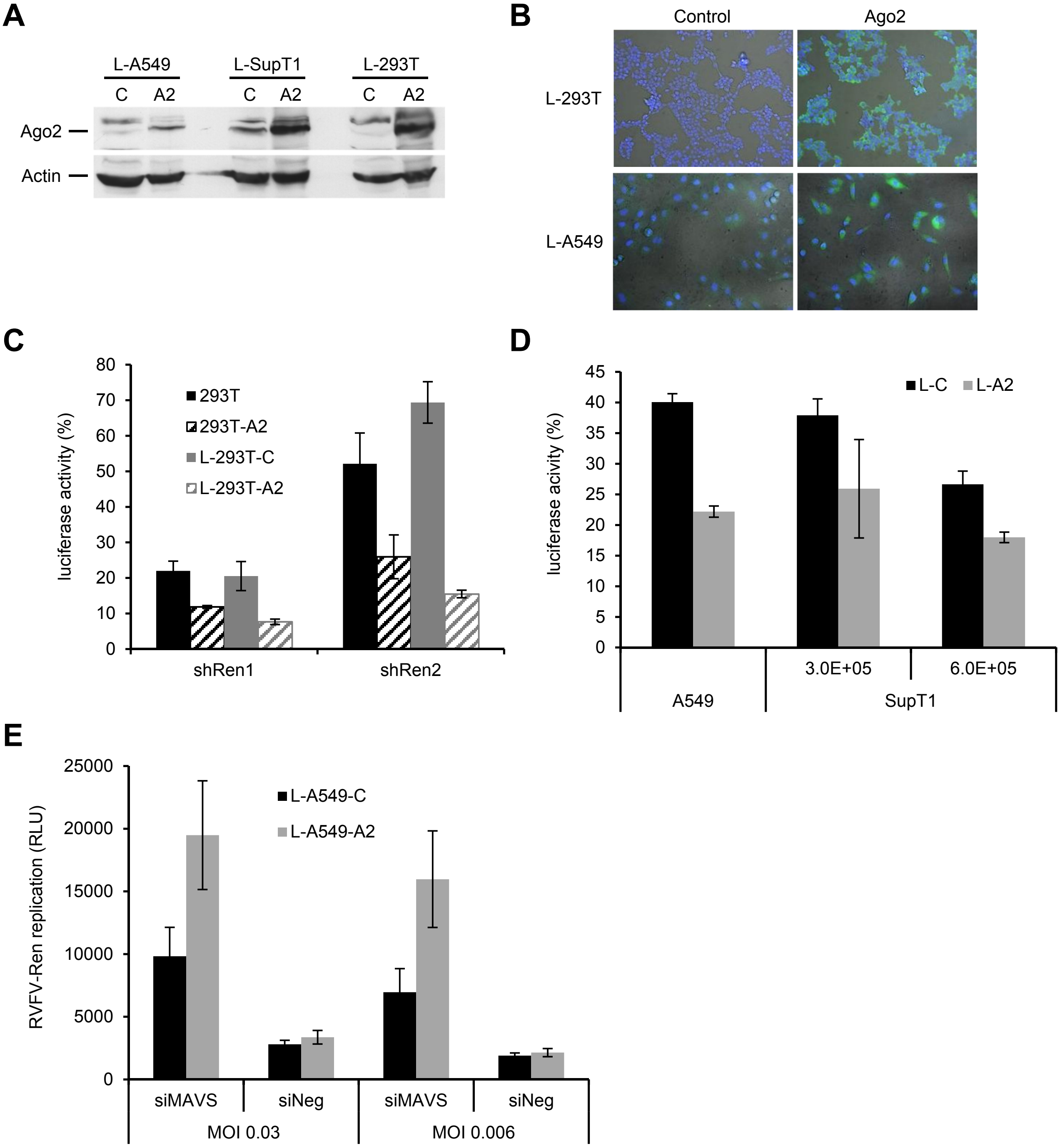

Börner *et al*. Supp Fig 5

SUPPLEMENTARY FIGURE LEGENDS

Supplementary Figure 1. Overview over cloning procedures to produce plasmids and AAV vectors co-encoding shRNAs and Ago2 (or Yfp as control). (**A**) Following replacement of two BbsI sites that we had originally used for shRNA expression from a U6 promoter with two BsmBI sites (step 1), the resulting cassette was merged with CMV promoter-driven Ago2 or Yfp (step 2). Step 3 consists of shRNA insertion as annealed oligonucleotides. Not shown is a derivative of plasmid pCA2n ("n" indicates the encoded neomycin resistance cassette) engineered to express blasticidin (hence named pCA2b) instead of neomycin. (**B**) Details of the shRNA cloning procedure in which two annealed oligonucleotides with 5' CACC or AAAA overhangs, respectively, are ligated to compatible BbsI or BsmBI ends (steps 1 and 3 as in A). (**C**) Translation of the Ago2/shRNA co-expression strategy into an AAV vector plasmid context (steps 1-2 show the cloning scheme for the Ago2 co-encoding vector, and steps 3-4 for the Yfp control). Steps 5-6 symbolize the final shRNA cloning as annealed oligonucleotides, using identical overhangs as in B. Fire, Firefly luciferase; polyA, polyadenylation signal; Ren, *Renilla* luciferase; SV40, SV40 early promoter; T6, terminator signal for U6 promoter; tk, thymidine kinase promoter. In the plasmid in the figure center, ITR, *rep* and *cap* are the AAV inverted terminal repeats, or replication and capsid genes, respectively. Plasmid p10825 was from Addgene (see Methods). Numbers in brackets after BbsI and BsmBI indicate the nucleotides (5'-3'/3'-5' strand) following the enzyme binding site where cleavage occurs.

Supplementary Figure 2. Additional validation 293T cells that Ago2 co-expression from an shRNA plasmid enhances RNAi. Shown is a comparison of luciferase knockdown efficiencies (normalized to the empty shRNA plasmid containing only the U6 promoter, set to 100%) with three different shRNAs expressed from a single shRNA/Ago2 plasmid (left), or from a separate construct that was co-transfected with the Ago2 expression plasmid (right). The cognate Yfp constructs were used as controls. Congruent with the data in Figure 2B, Ago2 mediated comparable improvements in RNAi when it was co-expressed *in cis* or *in trans*. Bars are means ± SD (n=3).

Supplementary Figure 3. Further characterization of stable Ago2 cell lines. (**A**) Growth behavior of parental HeLaP4 cells and four different stably Ago2-expressing derivatives. All cells were seeded equally (8000 cells per well in 96-well plates) and then counted regularly over three days. Clone D4 was chosen as one of our lead candidates based on its identical growth rates as compared to parental HeLaP4. The other three clones exemplify the different degrees of growth retardation typically observed. Except for clone B8 which exhibited the strongest Ago2 expression, Ago2 levels did not correlate with the extent of growth inhibition (data not shown). Values are means ± SD (n=5). (**B**) Representative results from miRNA profiling in stable Ago2 *versus* parental cells. Shown are dysregulated miRNAs in stably Ago2-expressing clones derived from HeLaP4 (left, delta value of 1.56) or 293T (right, delta value of 1.21). Up-regulated miRNAs are highlighted in red, down-regulated in green. (**C**) Repeat of the experiment from Figure 2F in Ago2-over-expressing HeLaP4 cells. Note that overall knockdowns were enhanced, and that competition between the two shRNAs was reduced, as compared to the parental cells in Figure 2F. Extra Ago2 expression from the different plasmids had marginal to no effects (especially at the higher doses of target-specific shRNAs), proving that Ago2 no longer rate-limits RNAi in the stable cells (see also D). Bars are means ± SEM (n=6). (**D**) Additional validation that Ago2 no longer restricts RNAi in stable Ago2 cell lines. Luciferase knockdown assays after co-transfection with psiCheck2 and either the single Ago2/shRNA construct or two separate shRNA and Ago2 plasmids showed the typical RNAi enhancement in normal Huh7.5 cells (left half). In contrast, the presence or absence of ectopic Ago2 made no difference in stable Huh7.5-Ago2 cells (right half). Bars are means ± SD (n=3).

Supplementary Figure 4. Analysis of dysregulation of cellular gene expression profiles upon siRNA delivery. Parental HeLaP4 cells (top) or the cognate Ago2-over-expressing cell line (bottom) were transfected with the siRNA against Myo10 (siMyo10) or an unrelated control siRNA (siCtrl). Changes in endogenous gene expression between the two siRNAs in each cell line were determined using a stringent fold change cutoff of $2^{0.5}$ (1.414) and a *P*-value cutoff of 0.01. As indicated by the dots and numbers, the total number of dysregulated genes was reduced from 460 to 268 in the Ago2-over-expressing clone. Moreover, there was a tendency towards milder alterations in the Ago2 cells.

Supplementary Figure 5. Generation of stably Ago2-expressing cell lines using a lentiviral vector. (**A**) Western blot depicting representative examples of Ago2 over-expression in three cell lines created using an Ago2-expressing lentiviral vector. Actin served as loading control. C, parental cells transduced with an empty control lentivirus; A2, Ago2-over-expressing clones. (**B**) Verification of Ago2

over-expression *via* anti-Ago2-immunofluorescence in two representative lentivirus-derived cell lines. A 10x objective was used for the images of the 293T cells, and a 20x for the A549 cells. Parental cells transduced with the empty control lentivirus are shown as controls. (**C**) Functional validation of the lentivirus-derived, Ago2-over-expressing 293T cell line (L-293T-A2) *via* transfection with psiCheck2 and the indicated shRNA plasmids. Luciferase knockdown was measured in direct comparison to parental 293T cells (293T), to cells generated through stable plasmid transfection (293T-A2, clone F6), and to control cells stably transduced with an empty lentiviral vector (L-293T-C). Luciferase values were always normalized to an irrelevant anti-hAAT shRNA control. Note that both stable Ago2 lines (hatched bars), derived through transfection or transduction, yielded similarly improved shRNA efficiencies (with a tendency towards stronger effects in the lentivirus-derived cells). Bars are means ± SD (n=3). (**D**) Further functional validation of the enhancing Ago2 effect in stably Ago2-encoding lentivirus-transduced A549 and SupT1 cells, compared to control cells (L-C) stably infected with the empty vector. Because both cell types are hard to transfect, we used an AAV vector (at the shown two different MOIs for SupT1) for potent Ren2 shRNA delivery. Bars are means ± SD (n=3). Luciferase values were normalized to an irrelevant anti-hAAT shRNA-expressing AAV. (**E**) Validation of lentivirus-derived Ago2 cells in a phenotypic viral infection assay. MAVS, an essential factor for the induction of the antiviral interferon system, was knocked down by siRNA transfection in lentivirally transduced A549 cells expressing Ago2 (gray bars) or an empty vector (black bars). Non-targeting siRNA served as negative control (siNeg). Cells were then infected with the shown two different MOIs of a reporter Rift Valley fever virus expressing *Renilla* luciferase (RVFV-Ren), and viral replication was measured by assessing luciferase activity 48 h post-infection. Ago2-expressing cells showed an approximately two-fold larger rescue effect upon MAVS knockdown than the cells transduced with the empty vector. Bars are means ± SD (n=3).

Supplementary Table 1. Summary of dysregulated genes in Ago2-over-expressing *versus* parental cell lines

Cell line	Gene symbol	Description	GenBank	Fold change
HeLaP4	*SSTR1*	Somatostatin receptor 1	NM_001049	-1.33
	CYHR1	cysteine/histidine-rich 1	NM_032687	1.33
	LOC652826	similar to 26S protease regulatory subunit 6B (MIP224) (MB67-interacting protein) (TAT-binding protein 7) (TBP-7)	XM_942509	1.51
293T	*SSTR1*	Somatostatin receptor 1	NM_001049	-1.65
	HEATR7B1	HEAT repeat containing 7B1	XM_291007	1.43
	IGDCC3	immunoglobulin superfamily, DCC subclass, member 3	NM_004884	1.47
	TRAPPC5	trafficking protein particle complex 5	NM_001042462	2.09
	TEAD4	TEA domain family member 4	NM_201443	2.97

Listed are exclusively those genes that were at least 1.33-fold dysregulated (up or down) in the Ago2-over-expressing lead clones (HeLaP4: D4, 293T: F6) as compared to their cognate parental cells. Note that very few candidates passed this threshold, exemplifying the largely maintained gene expression profiles in stably Ago2-expressing cells. Also note that somatostatin receptor 1 (SSTR1) was the only gene that was consistently down-regulated (for reasons unknown) in both cell types after stable Ago2 transfection.

Supplementary Table 2. List of image-based features (related to Figure 5F)

ID	Description
g.s	Geometry size
g.c	Geometry eccentricity
a.m.int	Morphology intensity (actin channel)
t.m.int	Morphology intensity (tubulin channel)
h.m.int	Morphology intensity (hoechst channel)
a.z.0101	Zernike moment 0101 (actin channel)
t.z.0101	Zernike moment 0101 (tubulin channel)
h.z.0101	Zernike moment 0101 (hoechst channel)
a.h.con	Haralick texture contrast (actin channel)
t.h.con	Haralick texture contrast (tubulin channel)
h.h.con	Haralick texture contrast (hoechst channel)
a.h.ent	Haralick texture entropy (actin channel)
t.h.ent	Haralick texture entropy (tubulin channel)
h.h.ent	Haralick texture entropy (hoechst channel)
a.h.asm	Haralick texture angular second moment (actin channel)
t.h.asm	Haralick texture angular second moment (tubulin channel)
h.h.asm	Haralick texture angular second moment (hoechst channel)
a.h.f12	Haralick texture information measure of correlation (actin channel)
t.h.f12	Haralick texture information measure of correlation (tubulin channel)
h.h.f12	Haralick texture information measure of correlation (hoechst channel)
at.cor	Correlation measure between actin and tubulin channels
ah.cor	Correlation measure between actin and hoechst channels
th.cor	Correlation measure between tubulin and hoechst channels

Detailed information about image analysis and feature extraction can be found in the Bioconductor package EBImage.
